# Critical review of adsorption technologies for heavy metal removal: mechanistic, computational, and policy perspectives

**DOI:** 10.1039/d6ra05305j

**Published:** 2026-09-16

**Authors:** Abdulrazzaq S. Abdullah, Akram A. Al-Asadi, Hassan Wathiq Ayoob, Haidar Hasan Mohammed, Raad Z. Homod, Hayder I. Mohammad

**Affiliations:** a Chemical & Petrochemical Techniques Engineering Department, Southern Technical University 61001 Basrah Iraq abdalmaliky@stu.edu.iq akramalasadi@stu.edu.iq; b Department of Chemical Engineering, University of Basrah 61004 Basrah Iraq hassan.wathiq@uobasrah.edu.iq; c Chemical Engineering Department, Universitat Rovira I Virgili 43003 Tarragona Spain; d Department of Chemical Engineering and Petroleum Refining, Basrah University for Oil and Gas 61004 Basrah Iraq haidar.alawaad@buog.edu.iq raadahmood@yahoo.com; e Department of Cooling and Air Conditioning Engineering, Imam Ja'afar Al-Sadiq University Baghdad Iraq hayder.i.mohammad@garmian.edu.krd

## Abstract

The main sources of heavy metal pollution in groundwater include discharges from petroleum refining plants, agricultural runoff, hospital wastewater, and environmental damage caused by war. Advances in adsorbents provide safe, efficient, and economical techniques for contaminant removal. This study presents a critical review of adsorption applications worldwide, particularly in Iraq, evaluating the effectiveness, large-scale applicability, and long-term sustainability of selected adsorbents. Furthermore, the study focuses on adsorption mechanisms, criteria for selecting appropriate adsorbents, process design, and advanced computational methods such as machine learning algorithms and molecular dynamics simulations. Biochar prepared from rice husk removes over 90% of Pb^2+^ under optimal batch conditions, while zeolite sourced from Samaraa removes more than 92% of Cr^6+^ and Cd^2+^. All machine learning models achieved prediction accuracies of more than 95% for adsorption performance. In addition, molecular dynamics simulations provide more insight at the atomic scale. This work addresses three primary problems: elevated salinity in the Euphrates river, ranging from 1000 to 3000 mg L^−1^; organic fouling resulting from effluent; and limitations imposed by existing infrastructure. In light of these conditions, the study recommends using advanced computational optimization models with mobile treatment units (MTUs) to provide solutions tailored to local conditions. The study further highlights the significance of pilot-scale testing and incorporation into policy frameworks. This helps close gaps related to Sustainable Development Goal 6 (SDG 6) on clean water and sanitation in Iraq, while also supporting environmental sustainability.

## Abstract 2

A critical review integrates adsorption mechanisms, computational methods, and scale-up strategies for heavy-metal removal, highlighting mobile treatment units and policy integration for sustainable water treatment in Iraq.

## Graphical abstract

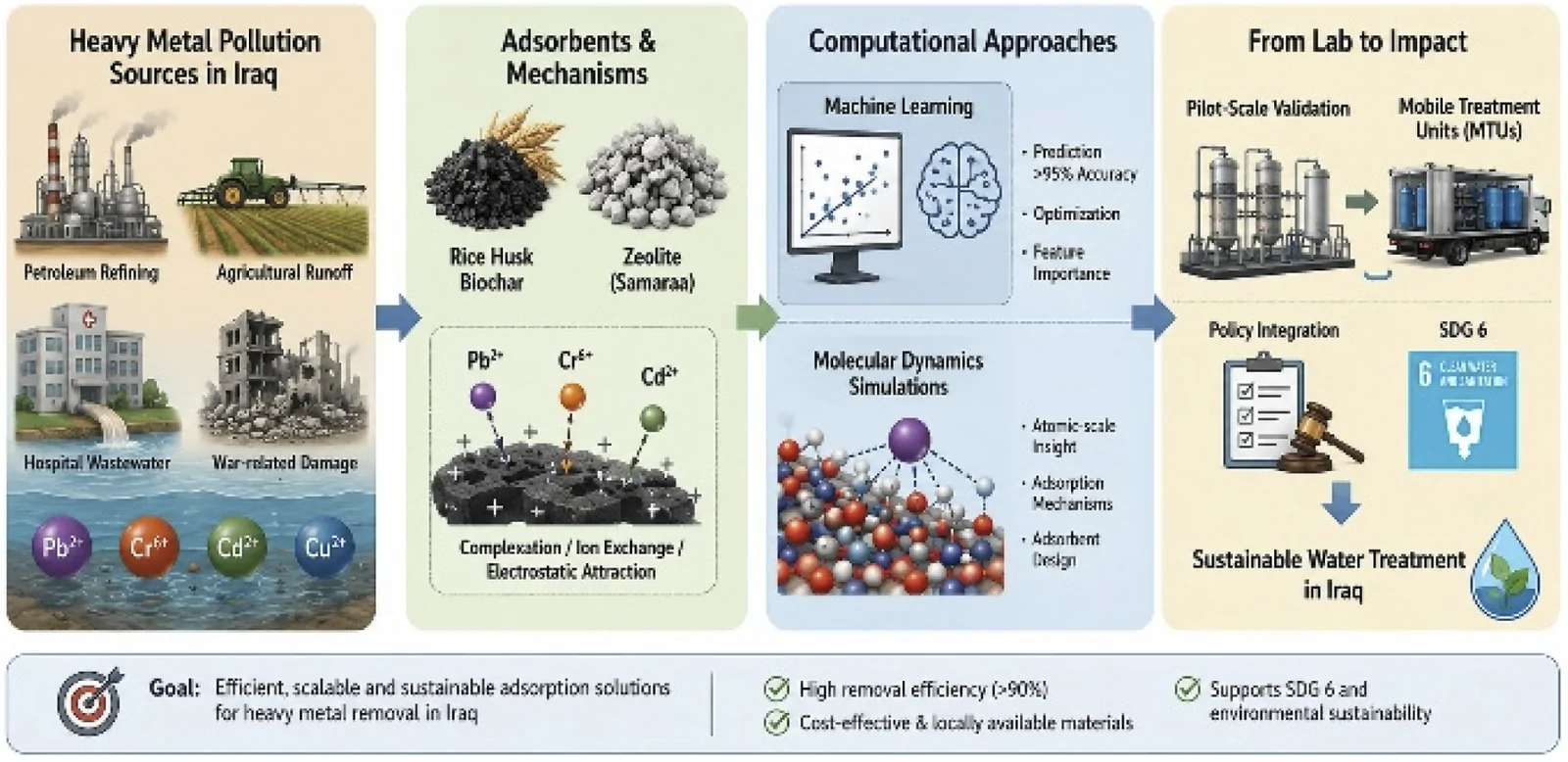

## 1. Introduction

Heavy metals pose a critical pollution threat to aquatic environments due to their persistence, potential for bioaccumulation, and toxicity. Among the most significant concerns are lead (Pb), cadmium (Cd), chromium (Cr), nickel (Ni), and arsenic (As).^1–5^ Industrial effluents, agricultural runoff, mining, and poorly managed urban discharges account for much of the contamination. The consequences can be devastating for aquatic environments, food security, and human health.^6–10^ In most practices, water treatment is defined as any strategy intended to remove contaminants resulting from anthropogenic activities before water is released back into nature or reused for a beneficial purpose. Among the methods proposed for water purification from contaminants, adsorption appears simple in process and feasible for efficient treatment, offering versatility where high-energy-requiring membrane filtration and electrochemical treatments, including reverse osmosis, cannot compete.^11–15^ Adsorption is described as “the attachment”, or “adhesion”, of molecules onto the solid surface of the adsorbent. An adsorbent refers to a solid material that has the ability to capture a particular component, whereas an adsorbate denotes a component or substance that can be trapped or adhered to the surface of the adsorbent. Several forces significantly govern adsorption, including van der Waals forces, hydrophobic forces, polarity, size exclusion, kinetic separation, steric interactions, and dipole-induced dipole interactions. Recent global studies recommend sustainable yet efficient adsorbents such as biochar, nanomaterials, agricultural wastes, clays, and zeolites, among new trend materials like graphene oxide and metal–organic frameworks (MOFs), with high removal efficiency (85–95% for Pb, Cd, Cr) together with environmental compatibility.^16–21^

It should be remembered that oil refineries, discharges from hospitals, agricultural activities, and war-related activities are the major immediate causes of heavy metal pollution in Iraq, which have eventually led to the contamination of clean sources such as the Tigris and Euphrates rivers, groundwater sources at Duhok and Shekhan, among other places, and industrial wastewater outlets in Basrah.^22–26^ Atomization of infrastructural damage during war and chemicals, especially in Mosul, caused an increase in the levels of Pb, Cd, and Cr above World Health Organization (WHO) standards.^27–31^ However, inadequate wastewater treatment infrastructure, with only approximately 30% of Iraq's population connected to sanitary sewer systems, significantly hinders remediation efforts and imposes substantial economic burdens.^9,32,33^ The country has abundant rice husks and date palm waste, including Samarra zeolites, which can be favorable for synthesizing eco-friendly, low-cost adsorbents with high potential for water purification.^15,17,34–37^

**Fig. 1:**
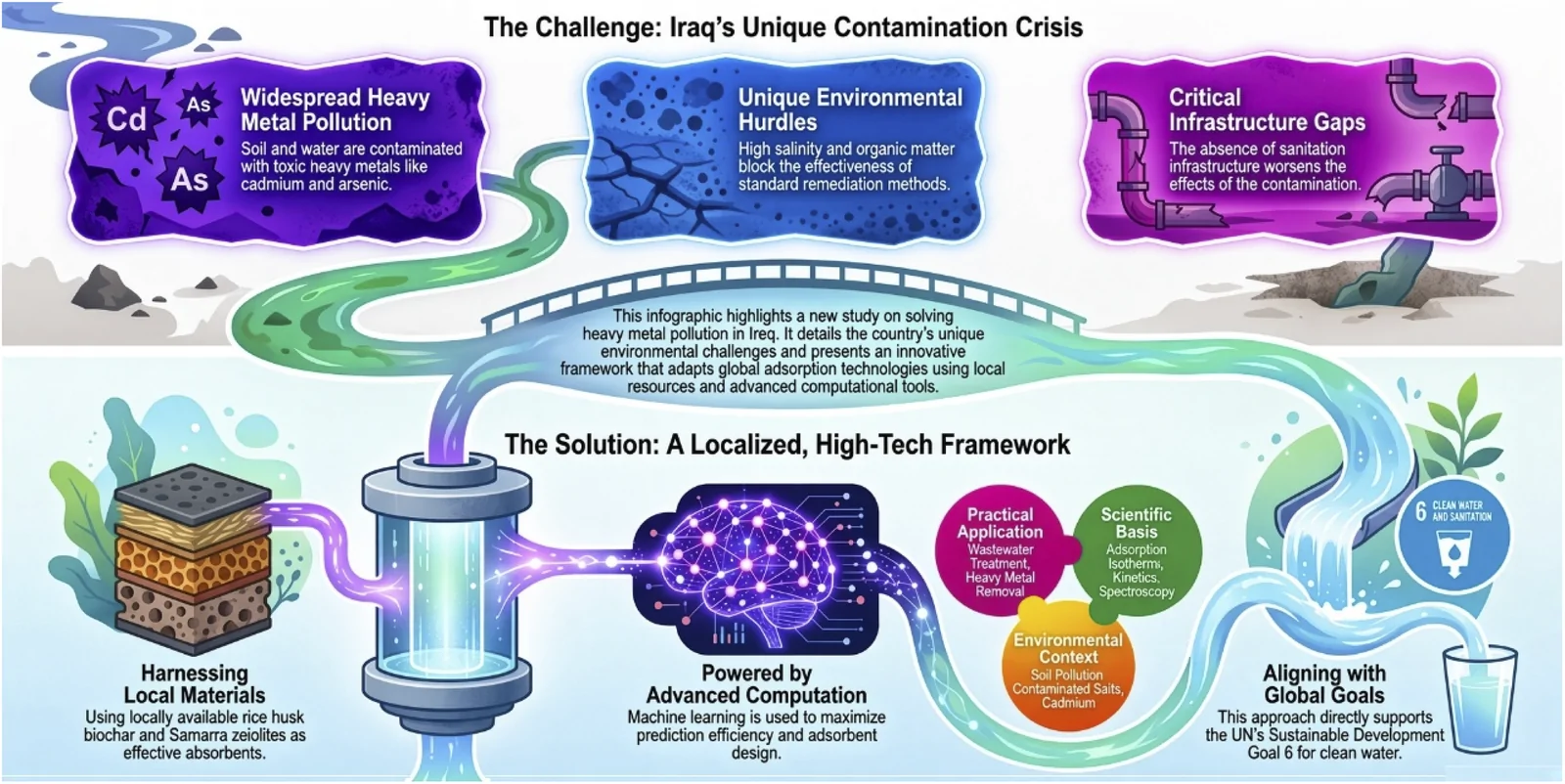
VOS viewer map illustrating the interconnections among research keywords related to adsorption, heavy metals, and environmental remediation in water and soil. Adapted from (ref. 2). Copyright © 2021, published by Elsevier B.V. Modification was made by the Gemini AI tool.

Fig. 1 visually presents the bonds in environmental remediation studies, with adsorption at the center. Node size indicates keyword frequency, and lines show their co-occurrence. In the blue cluster, adsorption is used for “wastewater treatment” and “heavy metal” removal from “contaminated water”. The green cluster provides the scientific basis for adsorption, including “adsorption isotherms”, “kinetics”, and analytical methods such as “Fourier transform infrared spectroscopy” for “heavy metal removal”. Most red clusters are related to “soil pollution” and “contaminated soils”, particularly regarding heavy metals-“cadmium and arsenic”-their remediation and bioavailability. This overview places adsorption as a practical approach for addressing heavy metal contamination in water and soil and highlights emerging research directions related to other problems.

This review addresses a major research gap in advanced technologies for heavy-metal remediation under Iraq's environmental conditions, which are salinity-sensitive, affected by hydrophobic organic blocking, and constrained by an inadequate sanitation system. Its main contribution is a detailed account of recent global progress in adsorption methods and their application in Iraq using locally available, low-cost adsorbents, including rice husk biochar and Samarra zeolites. Advanced computational tools, such as machine learning and molecular dynamics, are used to maximize prediction accuracy and to support adsorbent design. It also evaluates the effectiveness, scalability, and sustainability of these adsorbents as a cheap yet viable, pragmatic solution. Overall, this study holds significant value in its broad perspective and scope for tackling heavy metal contamination in regions facing substantial challenges. It contributes to environmental conservation at the local level and promotes global efforts toward clean water, aligning with Sustainable Development Goal 6 (SDG6) adopted under the United Nations Convention on water quality.

## 2. Literature search methodology

A systematic literature search was conducted to identify relevant studies on heavy metal adsorption, with a focus on sustainable, locally available adsorbents, biochar applications, and computational tools. The search covered papers published between 2018 and 2025 in the following databases and platforms: Wiley Online Library, Springer Link, Scopus, Web of Science, and Google Scholar. The search strategy combined main search terms with relevant keywords using Boolean operators. Main search terms included: groundwater remediation, biochar, computational tools, sustainable adsorbents, adsorption technologies, and heavy metal contamination. Only peer-reviewed articles published in English were included. Relevant studies were identified through backward and forward citation searches of key papers. The final literature search was completed in June 2026.

## 3. Heavy metal contamination in Iraq's liquid streams

Heavy metal contamination of water sources in Iraq, including the Tigris and Euphrates rivers, groundwater, and industrial effluents, poses an extreme environmental and public health crisis driven by a combination of anthropogenic and war-related activities.^22,24,26–28^ Key contaminants such as lead (Pb), cadmium (Cd), chromium (Cr), nickel (Ni), arsenic (As), and mercury (Hg) discussed earlier exceed WHO guideline levels, posing threats to ecosystems, agricultural productivity, and human health.^9,23,30,38^ Iraq faces a distinctive socio-environmental situation: high salinity, war-damaged facilities, and inadequate wastewater treatment systems combine to worsen existing pollution problems. This paper analyzes the sources of contamination and how they vary across regions, then considers the resulting socioeconomic and ecological effects. It also discusses health-risk evaluations and emphasizes the necessity for adsorption technologies customized to the complex composition of Iraqi wastewater.

### 3.1 Sources of heavy metal contamination

**Fig. 2:**
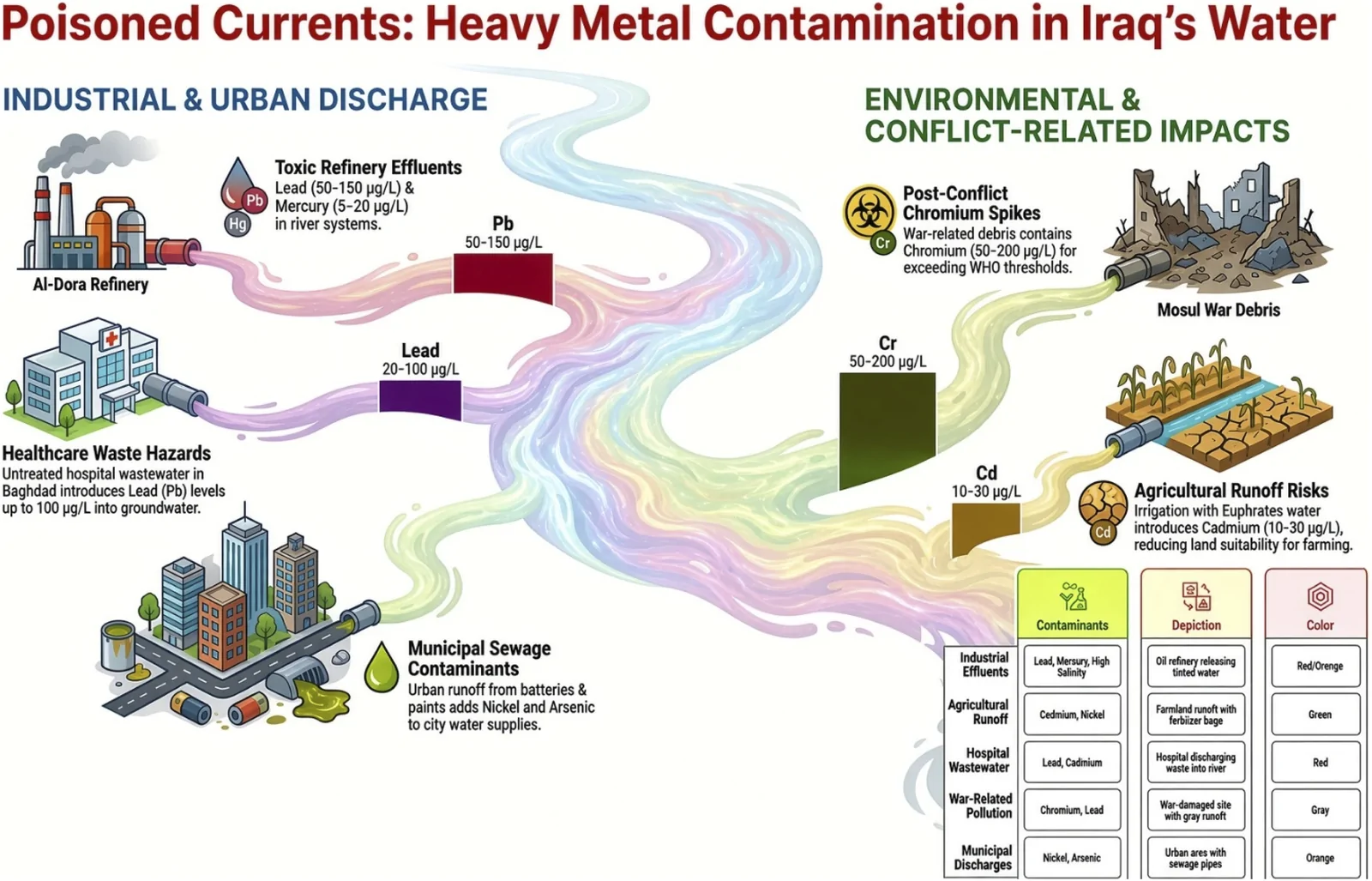
Sources of heavy metal contamination in Iraq. Adapted from (ref. 40). Copyright © 2022, published by Elsevier B.V. Modification was made by the Gemini AI tool.

Heavy-metal pollution in Iraq's water systems comes from several sources, each contributing different contaminants and creating distinct remediation challenges, as shown in Fig. 2.^9,22,24,26,28,31,39^ Industrial waste from oil refineries in Al-Dora and Basrah releases Pb, Cd, Cr, and Hg into the Tigris and Euphrates rivers. These wastewaters have high organic content and salinity, making treatment more difficult.^9,24,30,37^ For example, effluents from Al-Dora refinery contain Pb levels of 50–150 µg L^−1^ and Hg levels between 5–20 µg L^−1^, all above WHO standards.^31,43^ Agricultural runoff in areas like Duhok and Al-Hizamat has introduced elevated concentrations of Cd, As, and Ni, adversely affecting both groundwater and irrigation water quality. Specifically, Cd levels ranging from 10 to 30 µg L^−1^ have been reported in agricultural fields irrigated with water withdrawn from the Euphrates river, thereby lowering the suitability of the water for agricultural use.^25,29,44–47^ It is therefore imperative to develop adsorption technologies that can be customized and adapted to the actual contamination conditions in Iraq. Wastewater from hospitals and healthcare centers in Baghdad and Basrah adds more pollutants by discharging or largely untreated Pb, Cd, and Hg into the Tigris river and local groundwater. In Baghdad, Pb levels of 20–100 µg L^−1^ have already been found, which would be above the safe water standard and a source of major health risks.^5,15,23,26,39,48^ War-related contamination, especially in Mosul, raises concentrations of Cr, Pb, and Cd due to chemical spillage among other sources, further exacerbated by infrastructure implosion, where post-conflict debris in Mosul goes beyond WHO thresholds for Cr at 50–200 µg L^−1^.^27,41–43,46^ Additionally, poor sewage treatment in cities like Baghdad and Basrah allows Ni and As to enter the water from municipal sources, including batteries and paints, with urban runoff in Baghdad containing Ni (30–80 µg L^−1^) and As (5–15 µg L^−1^), further worsening water quality.^32,33,37,38,47,49^Fig. 2 shows where these sources are located in the area and which metals each one contributes. It highlights an urgent need for proper remediation.

### 3.2 Regional variations

Heavy metal pollution has significant regional variation in Iraq due to differential levels of industrial activity, agricultural practices, and conflict intensity. Hence, remediation strategies should be specific to each area, as these factors heavily influence the extent of contamination.^5,9^ Pollution in the Tigris river, which passes through Baghdad and Mosul, comes from hospital waste discharges and urban runoff, as well as debris related to war. In Baghdad, Pb levels vary between 20–100 µg L^−1^, while Cd levels vary between 5–30 µg L^−1^, which is just above WHO permissible limits, mostly attributed to hospital discharges and municipal wastes.^5,23,26,39^ War-related chemical spills and infrastructure damage in Mosul raise Cr values between 50–200 µg L^−1^ plus Pb between 50–150 µg L^−1^, posing very serious challenges to water quality.^27,43,47^ Additionally, the Euphrates river in Basrah is heavily contaminated with oil refinery wastes and agricultural wastes, in addition to high salinity (1000–3000 mg L^−1^),^24,30,31,40^ which lowers the efficiency of any applied remediation technologies. Consequently, remediation technologies are complicated by Pb levels between 50–150 µg L^−1^ and mercury (Hg) levels between 5–20 µg L^−1^ in Basrah city.^9,41^

Groundwater is mainly threatened by pollution through agricultural and industrial wastes. In these specific areas of Duhok and Shekhan, Cd levels between 10–30 µg L^−1^ and Ni concentrations between 20–50 µg L^−1^ have been recorded in wells in Duhok because fertilizers and pesticides, used abundantly, are the main contributors to the pollution.^25,29,44^ Groundwater contains 5–15 µg L^−1^, probably from pesticide application, further indicating water quality degradation in this particular area.^42,45^ The wetlands in the southern part are getting polluted mainly by industrial and agricultural wastes from upstream; particularly Pb and Cd are disturbing aquatic life and entering irrigation systems, creating a threat to biodiversity along with agricultural production.^44,46,49^ All these regional imbalances make a strong case for region-specific adsorption strategies as an effective solution to the heavy metal challenges that Iraq faces.

### 3.3 Socio-economic and environmental impacts

**Fig. 3:**
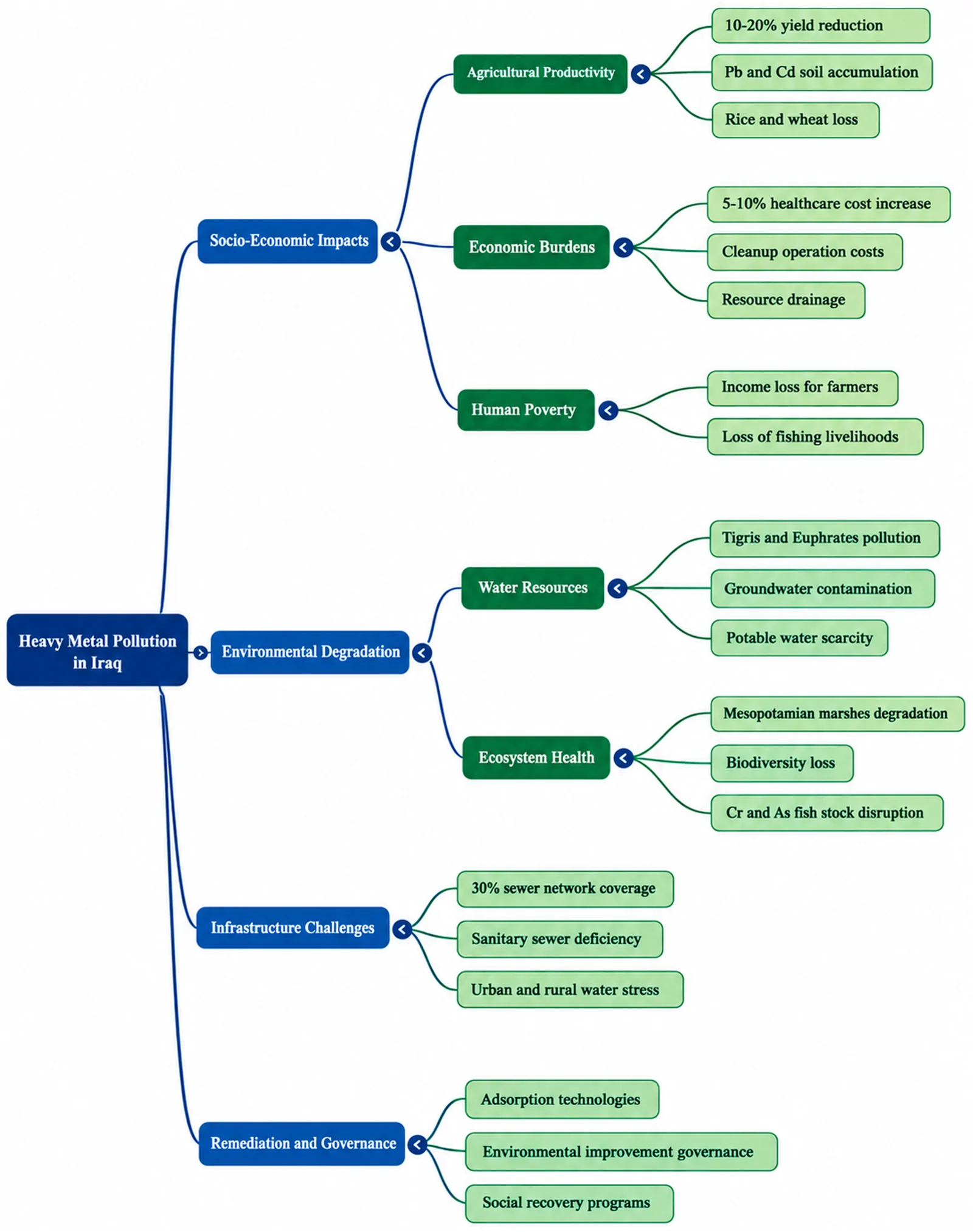
Socio-economic and environmental impacts of heavy metal contamination in Iraq. Adapted from (ref. 44). Copyright © 2023, published by Scientific Research Publishing. Modification was made by the Gemini AI tool.

Liquid stream heavy metal pollution in Iraq (the Tigris and Euphrates main rivers, groundwater, and wetlands) has direct impacts on the socio-economic and environmental levels that aggravate water scarcity, food insecurity, and economic burdens. Heavy metal irrigation in Basrah and Al-Hizamat reduces agricultural productivity by 10–20%, since this is the primary source of income for the population. Heavy metal pollution, combined with the fact that only 30% of the population is covered by sewer networks, intensifies water stress and biodiversity loss due to ecosystem degradation in Mesopotamian marshes. Moreover, it increases healthcare costs by about 5–10% in urban areas because of metal-related illness; thus, it drains Iraq's already limited economic resources. These impacts make a strong case for using adsorption technologies to remediate environmental damage by removing heavy-metal pollutants, while supporting social recovery programs grounded in environmental-improvement governance. Fig. 3 illustrates the interlinked impacts of water pollution across Iraq, highlighting its cascading effects on both socio-economic and ecological systems. Among the most severely affected sectors is the agricultural ecosystem, which experiences substantial environmental and productivity losses.^9,10,15,31,32,44^

The socio-economic and environmental effects resulting from heavy metal contamination in Iraq are the biggest obstacles to achieving sustainability and development in this region. Low agricultural productivity means reduced crop yields due to irrigating farmlands with contaminated water. In areas such as Al-Hizamat and Basrah, where lead (Pb) and cadmium (Cd) accumulate in the soil, these elements can directly affect rice and wheat production. Actual yield reductions in southern parts of the country brought about by levels of Cd in irrigation water were between 10 and 20%. The issue of water scarcity gets even worse because pollution happens on the Tigris and Euphrates rivers, from which potable water availability gets limited.^31,41,42,44,46^ In fact, only 30% of the population in Iraq is connected to sanitary sewers; hence, both urban and rural regions suffer more intensely from the problem of scarcity of clean drinking water.^28,32,47^ Heavy metal contamination also causes ecological degradation, in which aquatic ecosystems are highly disrupted and biodiversity decreases, for instance, in important zones like Mesopotamian marshes and the Tigris river system.^41,46,49^ For example, the accumulation of chromium (Cr) and arsenic (As) impairs fish stocks, causing misery and poverty among people living in these biomes.^42,44^ Furthermore, the costs associated with environmental remediation and the treatment of diseases caused by heavy metal exposure impose a substantial economic burden on Iraq. This raises the cost of medical attention by about 5–10% in towns due to exposure to lead and cadmium, which occurs in places that already have inadequate resources.^28,39,43^

### 3.4 Health risks

**Fig. 4:**
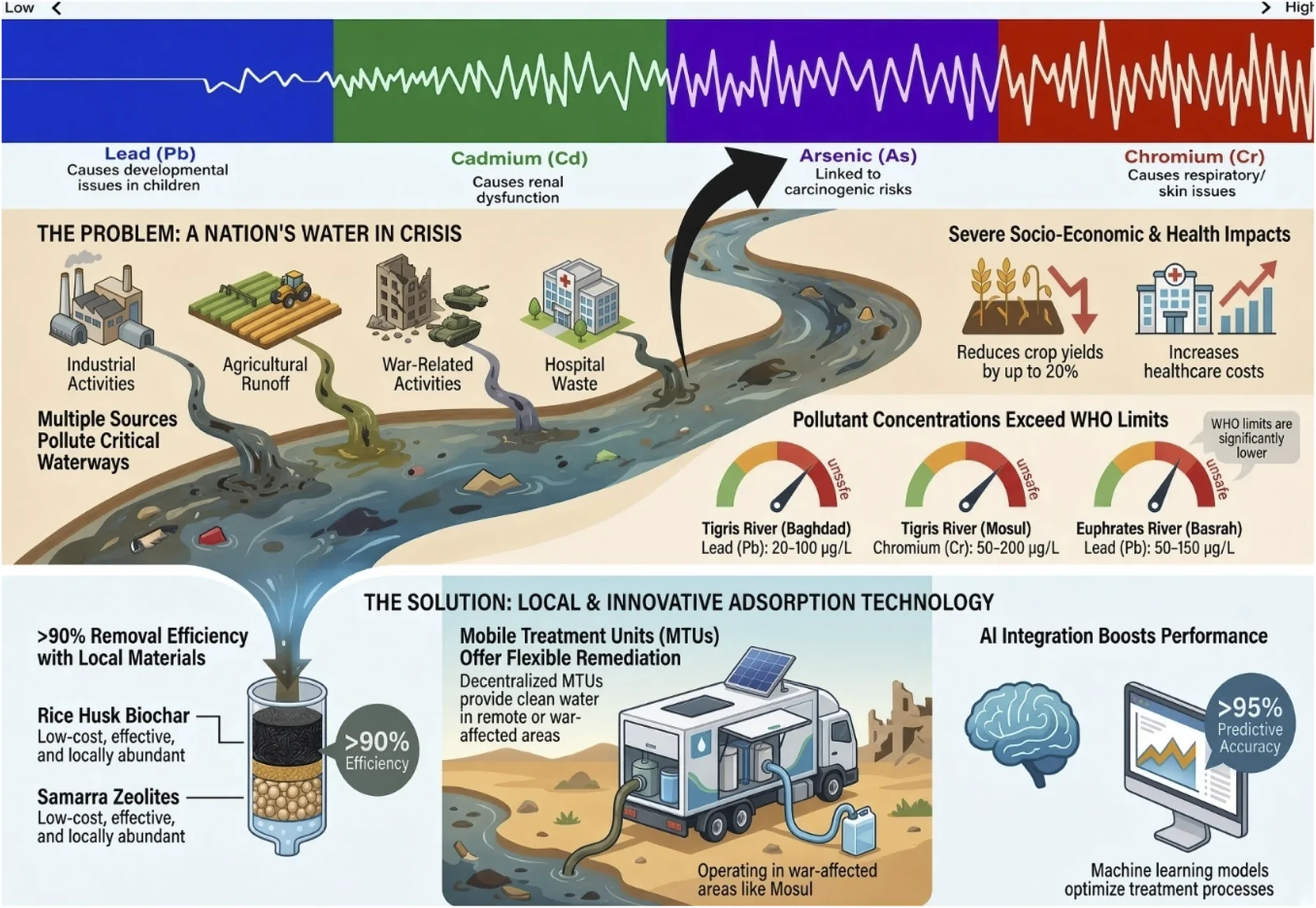
Health risks of heavy metal contamination in Iraq. Adapted from (ref. 27). Copyright © 2023, published by MDPI. Modification was made by the Gemini AI tool.

The ingestion of heavy metals through contaminated water and food chains in Iraq may have adverse effects on health and can result in diseases such as neurological disorders, nephrotoxicity, and carcinogenicity. Heavy metal levels in drinking water in Baghdad were found to fall within the range of 20–100 µg L^−1^, which is likely to cause growth problems in children. Groundwater from Duhok and Shekhan has Cd and As, which pose high risks of renal dysfunction and cancer. Mosul presents Cr contamination where chemical wastes of war cause respiratory diseases on one hand and skin diseases on the other. The above health effects underline the urgent need for practical remediation interventions through adsorption technologies to supply safe potable water and protect public health. Fig. 4 maps regions in Iraq affected by heavy metal contamination that pose a health risk, using color-coded fills to highlight areas, and links the country's different governorates to specific metals and associated health impacts that necessitate targeted remedial action.^5,23,25,26,39^

### 3.5 Implications for adsorption technologies

The water resources of Iraq are particularly challenging in the removal of heavy metals due to their high salinity, high concentration of organic matter, and often several heavy metal pollutants. Common adsorption processes are rendered ineffective in such complex waters, and hence, there is a need for adsorption techniques based on the composition of the water to be treated. Water pollution differs in character from one region to another in Iraq. For example, water resources in Basrah are contaminated with high salinity (1000–3000 mg L^−1^) due to seawater encroachment and decreased inflow of fresh water, while hospital and municipal wastewater in Baghdad carry substantial organic loads that could lead to adsorbent fouling and decreased adsorption efficiency. There is also localized pollution by chromium and lead in some areas of Mosul from industrial activities and the legacy of armed conflicts, as well as probably organic contamination within the hospital and municipal wastewater.

To overcome these challenges, much research has focused on using locally available adsorbents due to their low cost, sustainability, and accessibility. Agricultural by-products, rice husk biochar, date palm-derived biochar, and naturally occurring Samarra zeolites were selected for this study as they had shown promising potential for heavy metal removal under conditions relevant to Iraq. However, the choice of the adsorbent should take into account several variables: the salinity of the wastewater, its composition, competing ions, and the practical application to achieve effective and sustainable treatment. Decentralized treatment systems, including mobile treatment units (MTUs), may offer a practical way to provide potable water where conventional treatment facilities are damaged or absent. Integrated with suitable operating strategies and appropriate adsorption technologies, these systems could be especially suitable for emergency water treatment and humanitarian operations in conflict-affected areas. Table 1 summarizes the concentrations of heavy metals in Iraq's main aqueous systems, highlighting regional variations driven by different contamination sources. These findings provide the basis for discussing the adsorption technologies presented in the subsequent sections.^24,27,34,36,40^

**Table 1:** Heavy metal concentrations in Iraq's liquid streams

Region	Source	Pb (µg L^−1^)	Cd (µg L^−1^)	Cr (µg L^−1^)	Ni (µg L^−1^)	As (µg L^−1^)	Reference
Tigris river (Baghdad)	Hospital, urban runoff	20–100	5–30	10–50	30–80	5–15	23, 26 and 39
Tigris river (Mosul)	War-related actions	50–150	10–40	50–200	20–60	5–20	27, 43 and 47
Euphrates river (Basrah)	Oil refinery	50–150	5–20	20–80	10–50	5–20	24, 30 and 31
Groundwater (Duhok)	Agricultural runoff	10–50	10–30	5–20	20–50	5–15	25, 29 and 42
Al-Hizamat wetlands	Upstream discharges	20–80	5–25	10–40	10–30	5–10	44, 46 and 49

## 4. Adsorption mechanisms and technologies

Heavy metals (*e.g.*, Pb, Cd, Cr, As) from contaminated liquid streams in Iraq can generally be removed by adsorption, as this process is highly efficient, low-cost, and simple and versatile.^5,9,22,27,40^ This section discusses the mechanisms, materials, and process designs, in addition to specific applications to Iraq that will operate under conditions of high salinity and organic fouling.^10,15,50^

### 4.1 Adsorption mechanisms

**Fig. 5:**
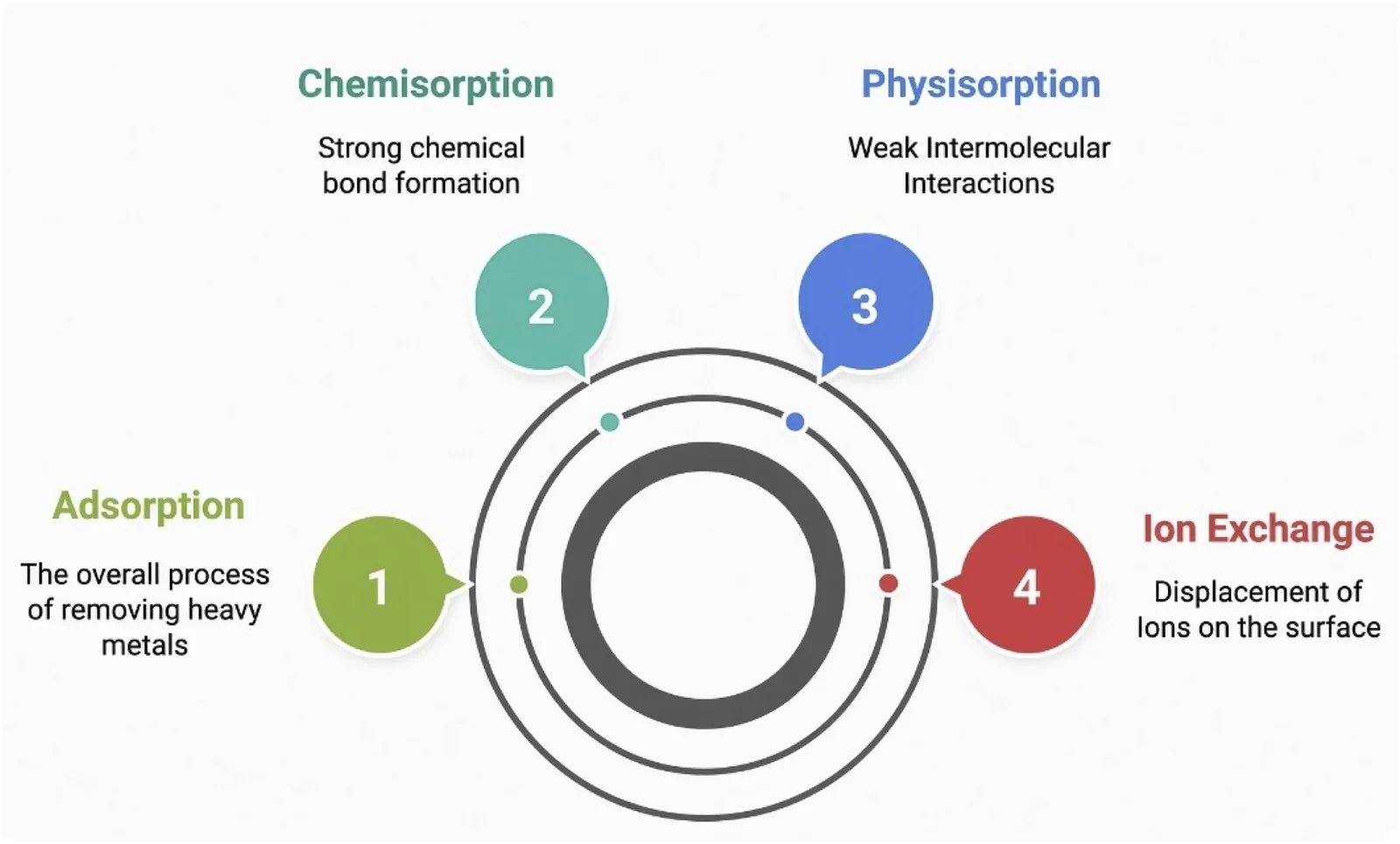
Schematic representation of adsorption mechanisms for heavy metal removal from wastewater.^34^ Adapted from (ref. 34). Copyright © 2024, published by Springer. Modification was made by the Gemini AI tool.

The three main mechanisms that govern adsorption for heavy metal removal in Iraq's wastewater treatment are determined by the nature of the wastewater and the properties of the adsorbent.^5,11–14^ Chemisorption relates to strong chemical bonds between metal ions (for instance, Pb^2+^, Cd^2+^) and any functional group (–OH or –COOH) of the adsorbents—biochar or clays—thus providing high selectivity and irreversibility.^11,14,15,33^ For example, more than 90% removal of lead (Pb) at a pH level between 5 and 6 has been found with biochar derived from rice husk using actual Tigris river water.^9,34^ This is quite advantageous since it demonstrates efficacy under conditions dominant in Iraq. van der Waals forces are weak; therefore, physisorption is used for adsorbents with a high surface area, such as activated carbon or nanomaterials, with binding strength described as lower in the organic-rich effluent.^4,10,12,50^ The second main mechanism associated with adsorption is ion exchange. In this process, cations (normally Na^+^) overtaking clay or zeolite sites can be exchanged by Pb^2+^ adsorption; since this wastewater contains several metals, this mechanism becomes predominant.^13,28,31,36^ Adsorption behavior can additionally be characterized in terms of kinetics, which most often fit pseudo-second-order models and isotherms such as Langmuir and Freundlich, with best performance at a pH of 5–6 and a temperature of 25–30 °C.^6,11,21,49^ High salinity in Iraqi wastewater reduces adsorption performance; therefore, optimization must be specific to enhance the process.^9,37,40^Fig. 5 focuses on these three mechanisms – chemisorption, physisorption, and ion exchange – demonstrating their validity for actual heavy metal removal from complex wastewater.^11,14,34^

### 4.2 Adsorbent materials

• Biochar from biomass pyrolysis. It shows high adsorption capacities (100–200 mg g^−1^ for Pb). This is mainly due to its porous structure and functional groups.^5,11,15,34,35^ On the other hand, date palm biochar works well for Basrah refinery effluents.^9,24^

• Nanomaterials: magnetic nanoparticles (Fe_3_O_4_) display fast kinetics with a good adsorption capacity falling in the range of 150 to 200 mg g^−1^ for Pb. However, synthesis entails high costs, and there is an environmental risk imposed.^4,10,16,33^

• Agricultural wastes: rice husk and pomegranate peels show adsorption capacities for Pb and Cd between 70 and 120 mg g^−1^, utilizing the agricultural resources of Iraq.^31,34,46,51^

• Clays: chemically modified bentonite yields between 60 and 90 mg g^−1^ for Cd and As adsorption. Notably, scalability is an issue due to processing costs.^6,7,28,39^

• Zeolites: Samarra zeolites show adsorption capacity between 80 and 110 mg g^−1^ for Pb, exhibiting excellent affinity for heavy metal ions.^5,36,37^

#### 4.2.1 Detailed physicochemical properties of promising adsorbents

The adsorption of any material is primarily governed by its physicochemical properties, including specific surface area, pore structure, surface functional groups, point of zero charge, and elemental composition. Adsorption mechanisms, such as electrostatic attraction, ion exchange, complexation, and pore filling, can be controlled by using these properties. They also affect adsorption capacity, kinetics, and selectivity toward heavy metal ions, especially in Iraqi wastewater. Such wastewater is typically polluted by organic fouling, high salinity, and changing pH. Low-cost sorbents that can be obtained locally are particularly attractive in Iraq. Date palm biochar, rice husk biochar, and natural zeolite from the Samarra region therefore show promising behaviors. Table 2 presents a comprehensive comparison of the main physicochemical properties of these three adsorbents, extracted from representative studies reported in the literature. These properties enable a clearer evaluation in the following discussion of their suitability for processing heavy metals under Iraqi conditions.

**Table 2:** Physicochemical properties of selected promising low-cost adsorbents for heavy metal removal (representative values from literature)

Property	Rice husk biochar (RH-BC)	Date palm/date pit biochar	Samarra/Iraqi natural zeolite
Specific surface area (BET, m^2^ g^−1^)	149 (pristine); up to 300–500 after chemical activation^34,37^	65 (moderate pyrolysis); 200–250+ at 600 °C or after activation (*e.g.*, ZnO or H_3_PO_4_)^35^	10–80 (typical for natural zeolites; increases with acid/heat treatment)^36^
Total pore volume (cm^3^ g^−1^) & distribution	Total ∼0.236; ∼23% micropores; ∼77% mesopores^34,47^	Total ∼0.08 (moderate); increases significantly with higher pyrolysis temperature or activation^11,35^	Predominantly microporous with uniform channels (0.1–0.3 typical)^36^
Elemental composition	High SiO_2_ (silica-rich), C, O; significant ash content^34,47^	High C, O, K, Ca, Mg; variable depending on feedstock part (pits *vs.* fronds)^35,38^	Aluminosilicate (Si, Al, O) with exchangeable cations (Na^+^, K^+^, Ca^2+^, Mg^2+^)^36^
Surface functional groups	Strong Si–O, O–Si–O; reduced O–H, C–H, C=O after pyrolysis^11,16^	O–H (hydroxyl), C=O (carboxyl), C–O, aromatic C=C^16,47^	Si–O–Al framework; surface hydroxyl groups^36^
Point of zero charge (pH_PZC)	9.6 (ref. 12 and 45)	6.5–8.0 (often alkaline)^35^	6.5–8.0 (ref. 36)
Particle size distribution	63–250 µm (common fraction used)^34^	Typically ground to <100–500 µm^38,47^	Crushed and sieved (0.5–2 mm granules or powder)^36^
Preparation and modification conditions	Slow pyrolysis (∼500–600 °C, N_2_ atmosphere); chemical activation with KOH or H_3_PO_4_ (ref. 34 and 47)	Pyrolysis of pits and fronds at 300–700 °C; common modifications include loading with ZnO or activation with phosphoric acid, both of which have been widely studied in Iraq^35^	Local mining/crushing/sieving; optional acid washing or thermal activation (400–550 °C)^36^

These physicochemical characteristics illustrate how the three adsorbents can work in a complementary manner. Owing to its high mesoporosity and silica-rich surface, rice husk biochar facilitates the rapid diffusion and mass transfer of metal ions. In contrast, date palm biochar is rich in oxygen-containing functional groups and can be obtained locally in Iraq, making it an economically attractive option. Samarra natural zeolite is notable for its high ion-exchange potential, which arises from its crystalline aluminosilicate configuration. Date pit biochar could, for instance, be further improved by combining it with ZnO. Zeolites can also be subjected to acid and thermal activation to increase the density of functional groups, extend their surface area, and enhance overall stability. This development would enable them to better resist the high salinity and organic fouling commonly encountered in Iraqi wastewater.

### 4.3 Emerging adsorbents

Recent advances in materials include graphene oxide, Metal–Organic Frameworks (MOFs) (a new category of crystalline porous substances), and bioinspired composite structures. All of these have been reported as promising adsorbents for the removal of heavy metals.^18–21,48^ Graphene oxide has demonstrated adsorption capacity of 200–300 mg g^−1^ for Pb, yet its high cost restricts practical application in the resource-constrained situations representative of Iraq.^15,20^ MOFs demonstrate strong selectivity in systems containing many metals and have been confirmed as suitable for treating real refinery effluents.^9,21,33^ Chitosan-based composite adsorbents encouraged by biological systems offer sustainable substitutes.^5,45,46^ These materials permit further testing at an experimental scale under the complex conditions of Iraq.^39,50^

### 4.4 Summary of key adsorbent types, performance, and applications

Table 3 summarizes the key categories of adsorbents and their applications, which are important and useful for water treatment in Iraq.^46^ It classifies the adsorbent types according to source or origin. It also lists their major advantages in Iraq in terms of the heavy metals they can possibly remove from water. Agricultural residues and Iraqi attapulgite clay are both locally available materials that can be obtained in a co-friendly manner and are capable of removing a wide range of heavy metals, ranging from lead and cadmium to some radioactive isotopes such as cesium-137.^3,50,51^ The application of widely available natural resources can be regarded as both environmentally friendly and cost-efficient, thereby opening a pathway for the progress of water purification technologies in the region.^31,50^ Furthermore, the table highlights advanced and purpose-designed adsorbents developed to achieve improved performance. One descriptive example is nano-activated carbon, which possesses high surface area and controllable properties that enable efficient removal of heavy metals including lead, cadmium, copper, nickel, and chromium.^9,15^ Others include more advanced materials like metal–organic frameworks (MOFs) and nanoscale zero-valent iron (nZVI), which have high porosity, tunable chemistry, selective adsorption, and removal efficiency toward various and complex heavy metal contaminants, for example chromium(vi), lead, and arsenic.^7,20^ In addition, biodegradable biopolymer-based adsorbents, as well as innovative hybrid and composite materials, exploit synergistic effects to provide broad-spectrum removal with easy separation, addressing complex industrial effluents while improving efficiency and environmental integrity.^2,8,35^

**Table 3:** Various adsorbent types, their origins, and their primary benefits for water treatment in Iraq

Adsorbent type	Origin/source	Key benefits for Iraq	Heavy metals targeted	Reference
Agricultural waste (*e.g.*, banana peels)	Local biomass, readily available agricultural by-products	Low-cost, sustainable, high surface area after activation	Lead, cadmium, various heavy metals	3 and 31
Iraqi attapulgite clay	Naturally abundant local mineral deposits in Iraq	Eco-friendly, low-cost, effective for specific radioactive isotopes and heavy metals	Cesium-137, various metal ions	50
Nano-activated carbon	Engineered from various carbon-rich precursors (*e.g.*, biomass)	Enhanced surface area, high adsorption capacity, customizable	Lead, cadmium, chromium, nickel, copper	9 and 15
Metal–organic frameworks (MOFs)	Synthesized porous coordination polymers	High porosity, tunable chemistry, selective adsorption, and high capacity	Diverse heavy metals, complex wastewater streams	20
Nanoscale zero-valent iron (nZVI)	Engineered iron nanoparticles	High reactivity, rapid removal *via* reduction and adsorption	Chromium(vi), lead, arsenic	7
Biopolymer-based adsorbents (*e.g.*, chitosan, alginate)	Derived from natural biopolymers	Biodegradable, environmentally friendly, good regeneration properties	Various heavy metals, especially in organic-rich waters	2 and 8
Hybrid and composite materials (*e.g.*, biochar + magnetite)	Combinations of different materials	Synergistic effects, enhanced selectivity, and easier separation	Broad spectrum of heavy metals, complex industrial effluents	9 and 35

### 4.5 Process design and optimization

Adsorption efficacy is related to process design between batch and fixed-bed systems, and thereafter with the optimization of operational parameters.^10,15,29,48^ While batch systems are good only for lab-scale studies, fixed-bed adsorption systems can be scaled up in wastewater treatment plants in Iraq.^28,43^ Spontaneity can be determined from kinetic studies (pseudo-second-order models) and thermodynamic analysis using Gibbs free energy, where the processes were found to be exothermic for Pb and Cd on biochar.^5,11,34^ Optimizing specific parameters, including pH (5–6), temperature (25–30 °C), and flow rate, can enhance treatment efficiency under high-salinity conditions.^9,37,40^

### 4.6 Hybrid reactor designs

Hybrids that associate adsorption with filtration or photocatalysis have been reported to be effective, particularly in complex matrices.^15,33,45,48^ For instance, adsorption-membrane systems have improved the removal of Pb and Cr from refinery effluents.^24,46^ Such systems should be further developed for the high-salinity and organic-rich wastewater of Iraq.^40,50^

### 4.7 Iraq-specific applications

The most effective adsorbents in this regard, which can be obtained locally and sustainably in Iraq, include rice husk biochar, date palm biochar, and natural zeolite from Samarra. Table 4 presents the results of the best-performing adsorbents. Rice husk biochar has high removal efficiencies for Pb (90–120 mg g^−1^). Date palm biochar performs very well for Pb (100–150 mg g^−1^) and may be applied in refinery effluents. Samarra zeolite has relatively high adsorption capacities for Pb (80–110 mg g^−1^) and can therefore help address pollution in regions like Mosul. It also presents these materials, along with modification strategies and their performance in actual Iraqi wastewater matrices. Such wastewater matrices are high in salinity and organic fouling and, therefore, the results emphasize the need for proper pre-treatment, such as coagulation–flocculation or desalination, to enhance adsorption capacity under local conditions.^24,34–36,50^

**Table 4:** Comparison of adsorbent capacities for heavy metal removal from wastewater

Adsorbent	Heavy metal	Adsorption capacity (mg g^−1^)	Optimal conditions	Cost	Scalability	Modification effect	Performance in real Iraqi wastewater	Iraq-specific notes	Reference
Rice husk biochar	Pb	90–120	pH 5, 25 °C	Low	High	Chemical activation (KOH/H_3_PO_4_) improves capacity	Effective removal reported in industrial wastewater; limited testing in high-salinity river water	Abundant in Iraq, effective for the Tigris river wastewater	34
	Cd	40–60	pH 6, 30 °C	Low	High	—	Adequate performance in low-to-moderate organic load conditions	Adequate for rural applications	34
Date palm biochar	Pb	100–150	pH 5, 25 °C	Low	High	ZnO loading significantly enhances capacity	Promising for refinery effluents; limited published data on real high-salinity conditions	Locally sourced, high potential for refinery effluents	35
	Cd	50–80	pH 6, 30 °C	Low	High	—	Limited data available for real Iraqi wastewater	Sustainable for Iraq's agricultural regions	35
Zeolites (Samarra)	Pb			Low	Medium	Acid/thermal activation improves performance	Successfully tested on real Tigris river water at Samarra; good removal efficiency	Effective for the Tigris river, locally available	36
	Cr			Low	Medium	—	Performance reduced in real river water due to organic fouling and ionic competition	Limited by regeneration challenges	36
Pomegranate peel	Pb	70–100	pH 5, 25 °C	Low	High	—	Limited testing in real Iraqi conditions	Viable for hospital wastewater in Baghdad	51
	Cd	30–50	pH 6, 30 °C	Low	High	—	Limited data available	Cost-effective for rural Iraq	51
Magnetic nanoparticles	Cr	150–200	pH 4, 25 °C	High	Low	Magnetic separation improves reusability	High efficiency in lab tests; very limited data in real Iraqi wastewater	High efficiency, but cost-prohibitive for Iraq	52
	Cd	100–150	pH 6, 30 °C	High	Low	—	Limited real-world application data	Limited scalability in Iraq	52
Activated carbon (date palm waste)	Pb		Batch adsorption conditions (optimized experimentally)	Low	High	Chemical activation of date palm waste significantly enhanced adsorption performance	Demonstrated high Pb removal efficiency (∼85%) in aqueous solutions; not evaluated using real Iraqi wastewater	Date palm waste is abundant in Iraq, making it a sustainable and low-cost precursor for activated carbon production	11
	Cr(vi)		Batch adsorption conditions (optimized experimentally)	Low	High	Chemical activation improved Cr(vi) adsorption performance	Demonstrated high Cr(vi) removal efficiency (∼85%) in aqueous solutions; not evaluated using real Iraqi wastewater	Suitable for converting Iraqi agricultural residues into value-added adsorbents for water treatment	11
Bentonite (modified)	As	60–90	pH 7, 25 °C	Medium	Medium	Requires modification for scalability	Limited data in real Iraqi wastewater	Effective for hospital wastewater	6
	Cd	40–70	pH 6, 25 °C	Medium	Medium	—	Limited real-world application	Requires modification for scalability	6
Chitosan-based composite	Pb	80–130	pH 5, 25 °C	Medium	Medium	—	Limited data available for real Iraqi conditions	Potential for complex wastewater matrices	53
	Ni	30–60	pH 6, 30 °C	Medium	Medium	—	Limited real-world data	Needs further testing in Iraq	53
Graphene oxide	Pb	200–250	pH 5, 25 °C	High	Low	—	Very limited testing in real Iraqi wastewater	High capacity, but impractical for Iraq	54
	As	100–150	pH 7, 25 °C	High	Low	—	Limited real-world application data	Cost limits large-scale use	54

#### 4.7.1 Real-world applicability of locally sourced adsorbents in Iraqi wastewater conditions

The successful real-world application of adsorption technology in Iraq depends greatly on the specific characteristics of local wastewater and the constraints of the existing facilities. Iraq's surface and groundwater, particularly the Euphrates and Tigris rivers and their branches, continue to face major threats. This is because of high organic loads, elevated salinity, and pollution from agricultural, industrial, and municipal sources. Under such conditions, it becomes difficult to maintain both adsorption performance and long-term sustainability. Basrah and areas associated with oil industry operations face particular challenges due to salinity. High salt concentrations reduce adsorption efficiency by competing ions at the interface and changing the surface charge of adsorbents. Removal efficiency tends to decrease with time when high organic content (mainly from untreated municipal and industrial discharges) causes organic pollution. Expanding adsorption applications in Iraq is further constrained by infrastructure challenges, including an inconsistent electricity supply, restricted access to advanced regeneration systems, and the necessity for minimal maintenance technologies. However, many locally available adsorbents have performed effectively under actual wastewater conditions.

Table 3 indicates that rice-husk biochar professionally removed lead from Tigris river water samples, whereas date-palm biochar showed extreme effectiveness for treating refinery wastewater in Basrah. High reduction in heavy metal concentrations was observed when testing a natural zeolite from Samarra with real river water. These materials are suitable for regions with limited infrastructure because they are inexpensive and locally available. Notably, modification strategies can potentially further improve their practical usability. Enhanced resistance to organic fouling and the ability to reuse the adsorbent can be improved by modifying date-palm waste-derived biochar with ZnO or activating zeolites through acid or thermal treatment. These changes are expected to preserve adsorption performance in complex matrices characterized by elevated salinity and high levels of organic fouling. However, under the conditions found in Iraq, treatment steps such as coagulation, flocculation, or partial desalination are generally desirable to achieve the highest adsorption effectiveness. The combination of locally available and appropriately modified adsorbents with suitable primary treatment therefore provides a practical way to treat heavy-metal pollution in Iraq. Although the laboratory results look promising, pilot-scale investigations under real-world field conditions remain necessary to confirm sustained performance and to determine whether it is worth the cost in Iraq.

#### 4.7.2 Economic aspects

Economic viability among the principal criteria used when selecting suitable adsorbents for wastewater treatment. This aspect holds particular significance in developing countries such as Iraq, where resources remain limited. An adsorbent that is locally available and inexpensive to produce would offer clear economic advantages. Table 5 classifies rice husk biochar and date palm biochar as inexpensive adsorbents because they are agricultural waste products. Natural zeolite sourced from the Samarra region represents another low-cost option, both in terms of acquisition and processing, and can serve as a practical alternative to expensive imported adsorbents such as activated carbon or graphene oxide. Beyond material cost, the ability to regenerate and reuse the adsorbent makes a real difference in the overall economics of the adsorption process. Specifically, biochar-based adsorbents generally regenerate effectively, helping to keep long-term operating expenses under control. Some adjustment techniques might add a bit to the initial cost, but they normally enhance performance and extend the life of the adsorbent, therefore improving economic efficiency in the long run. For instance, highly efficient materials such as magnetic nanoparticles and graphene oxide come with high adsorption capacities but also high production and regeneration costs, which makes them not feasible for large-scale application in Iraq. This is because the country has limited infrastructure support, high energy costs, and low accessibility to specialized regeneration facilities, therefore favoring a low-maintenance, locally available adsorbent. In general, rice husk biochar, date palm biochar, and Samarra zeolite are promising materials for removing heavy metals from wastewater in Iraq. Adopting them can reduce treatment costs while using local resources and supporting more sustainable waste management practices.

**Table 5:** Economic comparison of selected adsorbents for heavy metal removal

Adsorbent	Material cost	Regeneration cost	Reusability	Economic feasibility	Reference
Rice husk biochar	Low	Low	6–4	High	34
Date palm biochar	Low	Low-medium	5–4	High	35
Samarra zeolite	Low	Low-medium	5–3	High	36

## 5. Critical evaluation of adsorption technologies

As mentioned earlier, adsorption technologies can be effective and cost-efficient in the remediation of heavy metals in Iraq by using local raw materials-rice husk biochar and Samarra zeolites. They achieve over 90% removal for lead and cadmium, presenting sustainability options for contaminated water systems. However, organic fouling, scalability constraints, and performance decline under conditions of high salinity and matrix complexity pose obstacles to the hope articulated here that organic material resources could be found locally to address these issues. As shown in Table 4, the adsorbents were evaluated for removal efficiency, cost, scalability, and constraints in an Iraqi context. Therefore, based on the key findings, there is a need to optimize pre-treatment strategies and hybrid treatment systems to enhance adsorption performance.^5,34–36,50^

### 5.1 Advantages

Adsorbents used in the remediation of heavy metal-polluted water in Iraq also indicate some great advantages that make them very appropriate for the challenges regarding the treatment of water in this region. High efficiency leads to 85–95% removal rates of lead (Pb), Cadmium (Cd), and Chromium (Cr) under optimized conditions by Biochar and Zeolites, *etc.*, ensuring a quite strong mitigation method.^10,11,15,36^ Local materials such as rice husk and date palm biochar will significantly reduce the high cost of imported alternatives because there is an abundant supply of these resources from agriculture within Iraq.^17,28,51^ Sustainability is further supported by the fact that the adsorbent can be reused for roughly three to five cycles. Once spent, the material can still serve as a soil amendment, providing a green way to dispose of it and a practical means of resource recovery.^11,32,46^

### 5.2 Limitations

Although adsorption has significant advantages for water remediation in Iraq, a few challenges stand in its way, making it less effective. Organic-rich effluent, such as that from refineries and hospitals, causes fouling. This reduces adsorbent efficiency and therefore requires pre-treatment to achieve the best results.^4,33,50^ Another major challenge is scalability, as most adsorption studies are conducted in labs and pilot-scale validation is very limited; as a result, implementing them in practical scenarios becomes difficult.^36,47,48^ Additionally, high salinity and multi-metal systems from Iraq's wastewater matrix negatively impact adsorption performance, making treatment even more challenging.^31,37,40^

Biochar demonstrates higher adsorption capacities (100–200 mg g^−1^) compared with those of clays (60–90 mg g^−1^), thus proving a better choice for heavy metal removal.^5,6,36^ However, zeolites have the best ion exchange, particularly when applied in multi-metal systems, and wastewater from Iraq contains different waste compositions. Although nanomaterials exhibit very fast kinetics, they are not practical because the production route is expensive; hence, they cannot be used on large scales. On the other hand, MOFs fall under emerging adsorbents that seem promising; more investigations are necessary to optimize their adsorption capability to make them applicable in real-life, highly challenging wastewater conditions like those found in Iraq.^20,21^

### 5.3 Iraq-specific considerations

Local adsorbents offer significant advantages under the resource-constrained conditions prevailing in Iraq; however, fouling and high salinity remain important challenges that necessitate the development of hybrid treatment systems and the application of computational optimization techniques.^9,10,40,50^Table 6 provides a comparative evaluation of the performance of selected adsorbents for heavy metal removal from wastewater, based on their reported percentage removal efficiencies.

**Table 6:** Comparative evaluation of adsorbent performance for heavy metal removal from wastewater

Adsorbent	Material type	Heavy metal	Adsorption capacity (mg g^−1^)	Removal efficiency (%)	Regeneration potential	Cost (low/medium/high)	Scalability	Limitations	Iraq-specific applicability	Reference
Activated carbon (date palm waste)	Biomass-derived activated carbon	Pb		∼85		Low	High	Performance under highly saline wastewater was not evaluated	Produced from abundant Iraqi date palm waste, making it a sustainable and low-cost adsorbent	11
		Cr(vi)		∼85		Low	High	Regeneration and long-term reuse were not investigated	Suitable for utilizing agricultural residues for heavy-metal removal	11
Fe_3_O_4_ magnetic nanoparticles	Synthetic nanomaterial	Pb	130.2	98.5	High (desorption efficiency ≈98%)	High	Low	High synthesis cost; performance may be affected by nanoparticle agglomeration during practical application	High adsorption performance; however, large-scale implementation in Iraq may be limited by production cost and technical infrastructure	16
		Cd	57.0	93.6	High (desorption efficiency ≈98%)	High	Low	High synthesis cost; performance may be affected by nanoparticle agglomeration during practical application	Promising for advanced polishing of industrial wastewater but currently less feasible for large-scale deployment in Iraq	16
Zeolites (Samarra)	Natural mineral	Pb, Cr, Ni				Low	Medium	Adsorption capacity, regeneration potential, and optimization parameters were not reported	Locally sourced natural zeolite successfully evaluated using real Tigris river water from Samarra	36

## 6. Integration of advanced analytical and computational tools

Highly advanced computational tools include ML and molecular dynamics (MD) simulations that have begun to revolutionize the field of adsorption studies through predictive optimization capabilities and mechanistic insight.^55–59^ The wastewater matrices in Iraq are highly complex, with high salinity, organic fouling, and war pollution being some of the main characteristics.^24,27,28,40,50^

### 6.1 Machine learning approaches in adsorption studies

ML has become a valuable tool in adsorption research, largely because it can process complex, high-volume datasets, identify patterns that may otherwise remain hidden, and generate high-performance predictions. By handling multiple variables at once, these models generate estimates far more quickly than conventional isotherm and kinetic methods. This computational efficiency makes them particularly valuable for evaluation, fine-tuning, and optimizing adsorbents intended for heavy-metal removal from wastewater.

#### 6.1.1 Data sources and feature engineering

High-integrity data is essential for both developing machine-learning models and verifying their consistency. Researchers commonly construct their datasets from experimental results published in the literature, using public databases and prior studies to build the models. The quality of feature engineering also has a substantial influence on the model's performance. Usually, the most important elements are the physicochemical parameters of the adsorbent (surface area, pore volume, functional groups, *etc.*) along with the qualities of the adsorbate and the working conditions. By carefully selecting and properly preprocessing these variables, the predictive accuracy of ML models can be improved.^58^

#### 6.1.2 Model development and algorithms

**Fig. 6:**
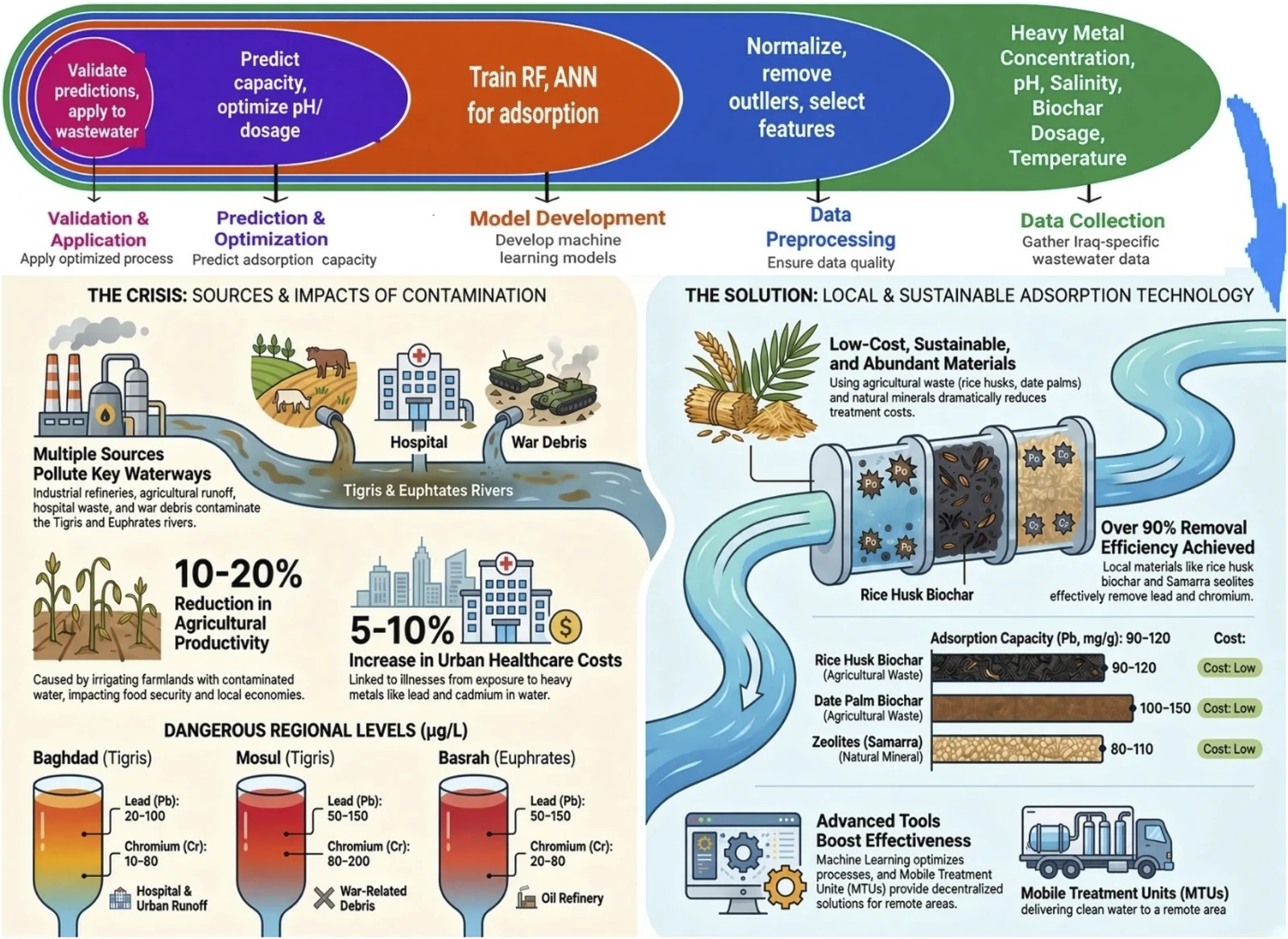
Machine learning workflow for optimizing heavy metal adsorption from wastewater. Adapted from (ref. 55). Copyright © 2023, published by Elsevier. Modification was made by the Gemini AI tool.

ML has been effectively applied to adsorption research. Artificial Neural Networks (ANNs), Random Forest, Support Vector Machines, and Gradient Boosting methods such as XGBoost are among the most often utilized techniques. These models can be employed to evaluate the removal efficiency and adsorption capacity. Ensemble approaches, particularly Random Forest and XGBoost, often perform well and generalize better when adsorption data are heterogeneous. Fig. 6 shows that machine learning models, including Random Forest and Artificial Neural Networks, can be trained effectively on locally relevant data to optimize heavy-metal removal from Iraqi wastewater through adsorption. The figure also illustrates how model development is integrated into a workflow with data preprocessing and performance validation.

#### 6.1.3 Prediction and optimization of adsorption performance

Machine learning models can predict adsorption capacity, selectivity, and kinetics under different operating conditions with sufficient accuracy. These models can therefore be used to quickly estimate the performance of a new adsorbent or to optimize process parameters without extensive laboratory experimentation by training the model on existing experimental data. Additionally, ML methods can be combined with optimization algorithms to find the best conditions for maximum removal efficiency, which is very useful in reducing the time and cost of experimental optimization.^18,57^

#### 6.1.4 Model validation, accuracy, and limitations

Reliability validation for ML models should be based on k-fold cross-validation and direct comparison of model predictions with experimental results. Typically, metrics such as *R*^2^ (coefficient of determination), RMSE (root mean square error), and MAE (mean absolute error) are used to assess model accuracy. Some key limitations regarding the applicability and reliability of ML models include the dependence on the quality and diversity of the training data for optimal performance. Another limitation is that most ML models provide predictions as “black boxes” without clear mechanistic insights; hence, to ensure robust and interpretable results, the predictions should always be validated experimentally and/or through mechanistic studies.^56,60^Table 7 shows the major limitations of current ML; therefore, the future of ML in heavy-metal adsorption should shift from accuracy-focused modeling toward standardized, physics-guided, and explainable adsorption informatics.

**Table 7:** Key limitations and future directions of ML in heavy-metal adsorption modelling

Limitation	Scientific consequence	Recommended solution	Reference
Data heterogeneity	Poor model transferability between independent datasets	Standardized adsorption databases	61 and 62
Different experimental protocols	Low reproducibility and biased model learning	Harmonized reporting of pH, ionic strength, dosage, contact time, temperature, and equilibrium criteria	61 and 62
Limited material descriptors	Weak mechanistic interpretation	Include BET area, pore volume, pH_PZC, zeta potential, functional groups, and elemental composition	63
Small datasets	Overfitting and unreliable extrapolation	Transfer learning, data augmentation, and cross-dataset validation	61
Black-box prediction	Lack of mechanistic insight	XAI methods such as SHAP, LIME, PDP, and feature importance	64 and 65
Lack of physical constraints	Physically unrealistic predictions outside training domain	PINNs incorporating kinetic, isotherm, diffusion, and mass-balance constraints	65 and 66
Incomplete material and adsorbate descriptors	Models may associate adsorption with operating variables while overlooking surface chemistry, pore architecture, mineral composition, or metal-specific properties, thereby weakening mechanistic interpretation and transferability	Include BET area, pore volume and distribution, surface functional groups, elemental/mineral composition, pH_PZC, zeta potential, particle size, surface morphology, metal speciation, and molecular descriptors	61, 63, 67 and 68

### 6.2 Molecular dynamics simulations in adsorption research

Molecular Dynamics (MD) simulation is a computational method used to track the movement of atoms and molecules over time. In adsorption studies, it offers detailed insights into how adsorbates interact with adsorbent surfaces at the atomic scale, a perspective that is often difficult to achieve through direct experimentation.

#### 6.2.1 Theoretical background and methodology

Molecular dynamics simulations are essentially based on the numerical solution of Newton's equations of motion for a particle system interacting through forces. Proper force fields and simulation parameters enable the modeling of the dynamic behavior of heavy metal ions on different adsorbent surfaces. Such an approach enables one to study the adsorption mechanisms under different environmental conditions, *e.g.*, varying pH, temperature, and ionic strength.

#### 6.2.2 Mechanism interpretation at the molecular level

Molecular dynamics simulations can help in understanding the adsorption mechanism at the molecular scale. The main interaction forces during adsorption can be identified by such simulations, *e.g.*, electrostatic forces, van der Waals forces, hydrogen bonding, and ion exchange. For instance, heavy metal ion sorption at the interlayer and nanopores of clay minerals has been studied by MD to obtain information on the role of surface functional groups and pore structure in the adsorption process.^69^ The adsorption of metal ions on functionalized silica-based materials has also been studied by MD to understand the enhancement of adsorption performance due to surface modification.^70^

#### 6.2.3 Limitations and computational challenges

It is essential to highlight that MD simulation has many limitations. The quality of the results obtained by MD is really the quality of the parameters of the force field used. In some cases, classical force fields cannot accurately describe complex interactions, such as chemical bonding or charge transfer; this can affect the reliability of the simulation results. Furthermore, MD simulations are very time-consuming, particularly if large systems or long timescales are involved. This could limit the technique's usefulness for high-throughput adsorbent screening. Therefore, MD is often combined with experimental studies and other computational techniques to validate and enhance its results.^71^

#### 6.2.4 Potential synergy with machine learning approaches

Although molecular dynamics offers insight into the underlying mechanism, it is computationally expensive and cannot efficiently screen a large number of materials. Combining MD simulations with ML approaches offers a way to overcome this restriction. Machine learning can support the analysis of large molecular dynamics datasets, enhance force-field development, and even forecast simulation results directly, which in turn minimizes computational costs. At the same time, molecular dynamics generates reliable, physics-based data that can support both the training process and the interpretability of ML models. Combining the two approaches can make adsorption studies both more efficient and more informative by linking machine learning's predictive strength with mechanistic interpretation from molecular dynamics.

### 6.3 Potential applications in the Iraqi context

Combining the ML and MD techniques can potentially advance heavy metal adsorption research in Iraq. As previously mentioned, industrial and agricultural wastewater in Iraq is characterized by high salinity, high organic loads, and pollution from industrial and farming activities.^22–24,28^ Computational tools can therefore support the refinement of treatment methods while reducing the need for extensive experimental testing. Machine learning models could also predict the adsorption performance of local materials, including rice husk biochar, date palm biochar, and natural zeolite from the Samarra region. Based on existing experimental data, these models can identify the conditions required to achieve maximum removal efficiency, reducing costly and time-consuming laboratory work. This advantage is particularly relevant in Iraq, where the considerable funding needed for large-scale experimental studies is often limited. MD simulations can further clarify, at the molecular scale, how heavy-metal ions attach to locally available adsorbents. A deeper comprehension of these interactions can guide material modifications, such as surface functionalization or the formation of composites. Such changes improve performance under real-world wastewater conditions characterized by high salinity and organic fouling.^36^

Pairing machine learning with molecular dynamics could further enable rapid screening and customized design of new or improved adsorbents suitable for Iraqi wastewater. In Iraq, however, the successful use of these advanced computational tools is impeded by several challenges, including restricted access to high-performance computing resources, a lack of comprehensive local datasets, and the requirement for specialized knowledge. Overcoming these challenges by strengthening research capacity and fostering collaboration will be essential for Iraq to realize the greatest benefit from machine learning and molecular dynamics. Overall, integrating these computational tools could help create more effective, lower-cost, and sustainable methods for heavy-metal remediation in Iraq, thereby strengthening environmental management and safeguarding public health.

### 6.4 Challenges and future perspectives

**Fig. 7:**
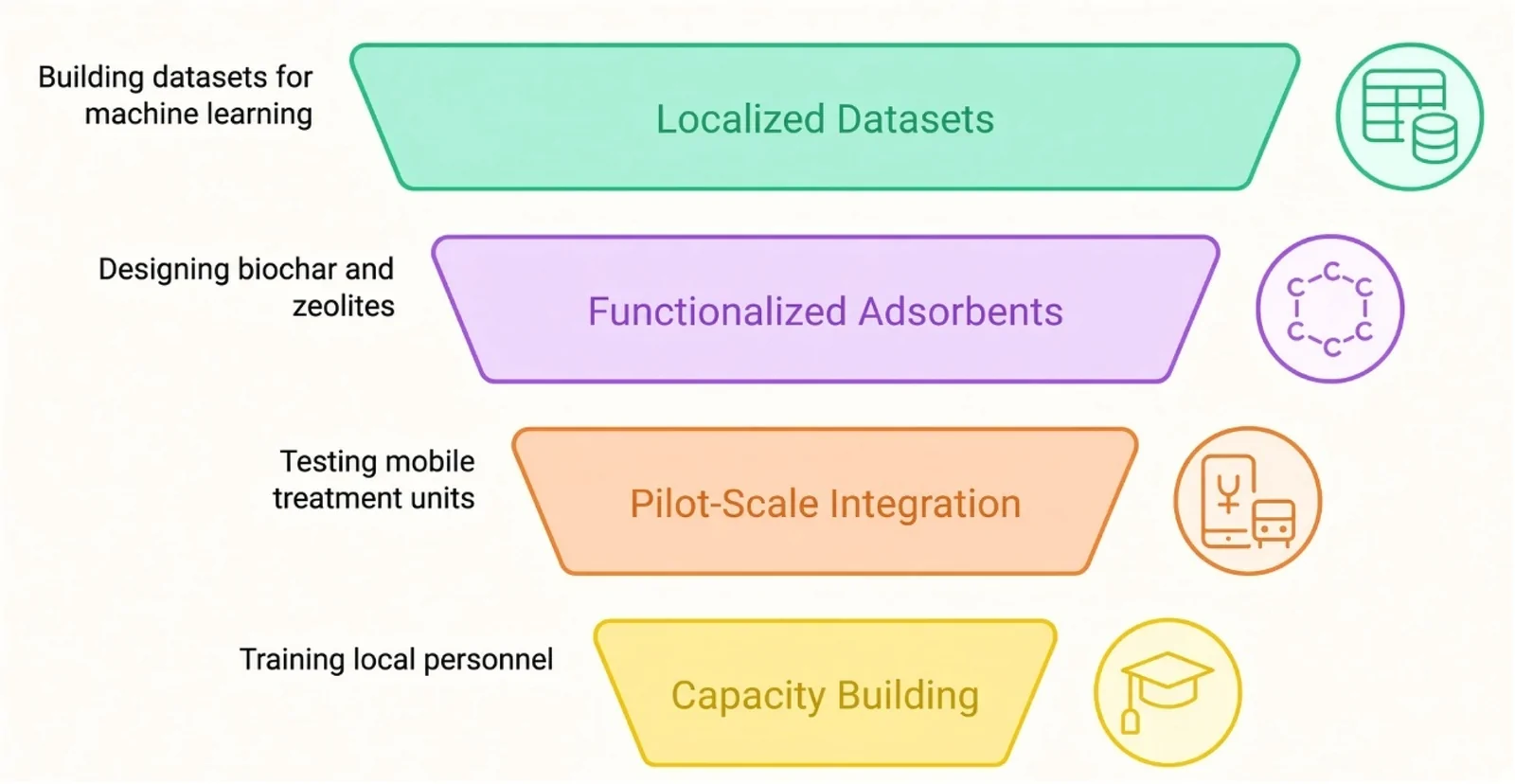
Future directions for adsorption in Iraq. Adapted from (ref. 55). Copyright © 2023, published by Elsevier. Modification was made by the Gemini AI tool.

Although ML and MD are being used more often in adsorption studies, applying them in Iraq remains difficult. Local datasets are scarce, computational infrastructure is inadequate, and the shortage of expert manpower limits the development of reliable models.^55,56^ Validating computational predictions against experimental results is also challenging. Few local studies examine actual wastewater conditions, especially high salinity and organic fouling.^22,24,28^ To help in overcoming these constraints, several opportunities are seen in the future. As shown in Fig. 7, future steps to advance adsorption technology in Iraq include developing localized datasets for machine learning, functionalized adsorbents, pilot-scale treatment units, and a capacity-building program. This will be a major step in enhancing the predictive model's performance due to the localized nature of the data. The accuracy and reliability of the model can be further improved by integrating ML and MD with experimental studies. A hybrid computational framework that will be developed in due course will combine the predictive capability of machine learning with the mechanistic insight of molecular dynamics to deliver more efficient and sustainable wastewater treatment solutions in Iraq.

### 6.5 Advanced computational chemistry for heavy-metal adsorption: from static DFT calculations to multiscale molecular simulations

Density Functional Theory (DFT) has become one of the major computational techniques for studying heavy-metal adsorption because it provides molecular-level information that is not easily accessible through experiments. In addition to calculating adsorption energies, DFT can identify favored adsorption sites, charge redistribution, electronic interactions, and the development of surface complexes between metal ions and functional groups. However, the trustworthiness of these predictions is closely related to the computational technique chosen, in particular the choice of exchange–correlation functional, basis set, effective core potential, dispersion correction, and solvent representation.^72,73^

A major shortcoming in many published DFT investigations is the simplistic representation of the aquatic environment. To lower the computing cost, most calculations are based on implicit solvation models, such as the Polarizable Continuum Model (PCM), Conductor-like Screening Model (COSMO), or Solvation Model based on Density (SMD). These approaches describe water as a homogeneous dielectric medium and usually give reasonable estimates for bulk solvation effects. However, they do not explicitly account for hydrogen-bond networks, hydration shell rearrangement, proton transfer events, or the constant contact between water molecules and the adsorbent surface. As a consequence, the adsorption geometries and adsorption energies derived from implicit models can vary from those expected for true aquatic conditions. Therefore, considerable differences in the projected adsorption behaviors can arise for the same adsorbent-metal system under different computational parameters. For this reason, comparisons between separate DFT studies should always be made taking into account the computational technique and not only the adsorption energy. One has to be very careful with the basis set, since heavy-metal ions are characterized by strong relativistic effects, which are important for their electronic structure. Treating all electrons explicitly is typically computationally expensive and does not necessarily improve forecast accuracy. Therefore, effective core potentials (ECPs) are generally used to replace chemically inactive core electrons while maintaining an explicit description of the valence electrons important for adsorption.

LANL2DZ, among the available ECPs, is still extensively utilized, which gives a more practical compromise between computational efficiency and acceptable accuracy. However, Stuttgart–Dresden (SDD) pseudopotentials usually offer a more rigorous explanation of scalar relativistic effects and are hence preferable for adsorption systems containing Pb, Cd, Hg and related heavy metals where better accuracy is desired. Consequently, the choice of an ECP should be determined by the features of the adsorption system and the aims of the computation, rather than by a single standardized methodology.^74^ Another significant source of variation in DFT predictions is the choice of the exchange–correlation functional. The most widely used generalized gradient approximation (GGA) functional for the study of adsorption involving periodic materials such as metal oxides, zeolites, graphene derivatives, and metal–organic frameworks remains Perdew–Burke–Ernzerhof (PBE), as it provides a reasonable compromise between computational efficiency and structural accuracy. However, standard PBE does not capture long-range dispersion interactions correctly, which are often important in the adsorption of hydrated heavy-metal species. Thus, dispersion-adjusted methods like PBE-D3 often yield more accurate adsorption geometries and adsorption energies for environmental systems.^75^

Hybrid functionals are often chosen when a better description of the electrostatic interactions is desired. B3LYP has been widely used for adsorption investigations due to the inclusion of the accurate Hartree–Fock exchange to better describe charge transfer and metal–ligand interactions. However, its computational cost increases significantly with system size and is therefore better suited for cluster models than for large periodic structures. On the other hand, the M06 family has received growing attention for adsorption involving transition metals, since it offers a superior treatment of medium-range electron correlation and non-covalent interactions. As a consequence, M06 has been effectively applied to systems with Pb(ii), Cd(ii), Hg(ii), Cr(vi), and As(v), where the adsorption behavior is controlled by both coordination chemistry and weak intermolecular interactions.^76,77^

It is worth noting that DFT provides useful information on equilibrium adsorption structures, while conventional calculations describe only static arrangements. In contrast, explicit-solvent models include individual water molecules in the simulation and consequently consider hydration effects, hydrogen-bond rearrangement, competitive adsorption, and solvent-mediated interactions not considered by continuum models. The importance of these effects increases as adsorption occurs in complicated aquatic environments with competing ions or dissolved organic matter. Classical Molecular Dynamics (MD) complements DFT by studying the time-dependent behavior of hydrated metal ions near solid surfaces. Therefore, in circumstances closer to practical water-treatment systems, diffusion, adsorption–desorption events, solvent restructuring, and temperature effects can be studied. This approach is generalized in *Ab initio* Molecular Dynamics (AIMD) by combining atomic motion with electronic-structure calculations, allowing transient adsorption intermediates, proton-transfer reactions, and surface hydroxylation processes to be investigated without relying completely on empirical force fields.

The combination of various computational methodologies yields complementary rather than competing information. DFT is well-suited to locate adsorption sites and estimate binding energies, and MD and AIMD depict the dynamic mechanisms that regulate adsorption under realistic aquatic conditions. Specifically, the combination of the two techniques, along with spectroscopic characterization and adsorption tests, provides a stronger basis for elucidating adsorption mechanisms and driving the design of next-generation adsorbent materials. Tables 8 and 9 summarize representative computational research and the dependence of adsorption predictions on the computational framework chosen. Due to the decreased computing cost, most conventional DFT investigations still adopt vacuum or implicit-solvent models.

**Table 8:** Representative computational studies on heavy-metal adsorption and solvation treatment

Adsorbent	Metal ion(s)	Computational framework	Solvation treatment	Major findings	Critical limitation	Reference
Graphene oxide	Pb^2+^, Cd^2+^	DFT-based analysis/review	Mostly idealized or implicit	Oxygen-containing groups strongly control Pb/Cd adsorption	Hydration-shell dynamics are not fully resolved	78
Graphene/graphene oxide (GO)	Pb^2+^, Cd^2+^	Periodic DFT, PBE0	No solvent considered	GO showed stronger interaction than pristine graphene	Explicit water and competing ions were neglected	79
Functionalized CNT/BNNT	Pb^2+^	MD + predictive modelling	Explicit water/ions	Functional groups enhanced Pb adsorption; RDF and interaction energies clarified adsorption behavior	Electronic charge transfer was not captured because DFT was not used	80
Zeolite frameworks	Pb^2+^, Cd^2+^	Monte Carlo + MD + ANN	Explicit aqueous modelling	Molecular simulation and ANN were combined to screen zeolite performance	Electronic-level surface complexation was not described	81
MOF system	Cd^2+^	DFT solvent-effect study	Solvent-dependent modelling	Solvent effects significantly influenced Cd-MOF adsorption interactions	Does not fully reproduce complex wastewater matrices	82
Graphene nanochannel	Pb^2+^, Cd^2+^, Hg^2+^	Classical MD	Explicit water/electric field	Ion accumulation and transport were affected by electrostatic interactions	Focused more on transport than equilibrium adsorption	83
ZnO nanotube/ZnO sheet	Ni, Cu, Cd, Ag	DFT	Vacuum	Preferential adsorption sites and adsorption energies were evaluated	Solvent and competitive adsorption were neglected	84
Functionalized SWCNT	Heavy-metal ions	DFT	Idealized molecular model	Surface functionalization improved metal-ion binding	Real aqueous chemistry was not explicitly represented	85

**Table 9:** Critical comparison of computational frameworks for solvation and surface complexation

Computational framework	Explicit solvation	Implicit solvation	Surface complexation	Hydration-shell description	Competitive ions	Computational cost	Recommended use	Reference
Static DFT under vacuum	Not included	Not included	Good for isolated binding sites	Not captured	Not captured	Low-medium	Initial screening of adsorption sites	76 and 86
Periodic DFT + implicit solvent	Not molecularly explicit	Included as dielectric continuum	Moderate	Poor	Poor	Medium	Estimating adsorption energies on extended surfaces	73 and 87
DFT-D3	Usually implicit or vacuum	Possible	Improved for weak interactions	Poor unless explicit water is added	Poor	Medium	Systems where dispersion affects adsorption	75 and 88
Hybrid DFT, *e.g.*, B3LYP	Possible in cluster models	Possible	Strong electronic-level description	Limited	Limited	High	Cluster models and charge-transfer analysis	74 and 76
Meta-hybrid DFT, *e.g.*, M06	Possible in cluster models	Possible	Strong for transition-metal interactions	Limited-moderate	Limited	High	Heavy-metal complexes and functional groups	77 and 89
Classical MD	Fully possible	Not typical	Indirect; force-field dependent	Strong	Strong	Medium-high	Hydration, diffusion, and competitive adsorption	80 and 81
AIMD	Fully possible	Possible but less preferred	Excellent	Excellent	Possible but expensive	Very high	Proton transfer, hydroxylation, and realistic interfacial adsorption	72 and 90

## 7. Challenges and opportunities in Iraq

High salinity, organic fouling, war-induced pollution, and infrastructural inadequacies are major challenges in adsorption technology that can be addressed simultaneously, with an opportunity for innovative solutions in Iraq's complex socio-environmental setting. Some varieties, such as rice husk biochar and zeolites from Samarra, are recognized as locally produced adsorbents, offering a sustainable alternative. For some, household-level decentralized remediation options using Mobile Treatment Units (MTUs) are suitable for war-related situations such as those in Mosul. Other factors that could be considered as adjustments are pre-treatment adaptive to computational optimization aimed at reducing the impacts of salinity and fouling. The major challenges hindering wastewater treatment in Iraq, including high salinity, inadequate infrastructure, and other operational constraints, are critically assessed alongside the potential benefits of emerging approaches, such as mobile treatment units (MTUs) and locally available adsorbents. This analysis identifies promising strategies for addressing these barriers and enhancing the efficiency, reliability, and practical applicability of adsorption-based wastewater treatment technologies in Iraq.^27,28,32,40^

### 7.1 Challenges

**Fig. 8:**
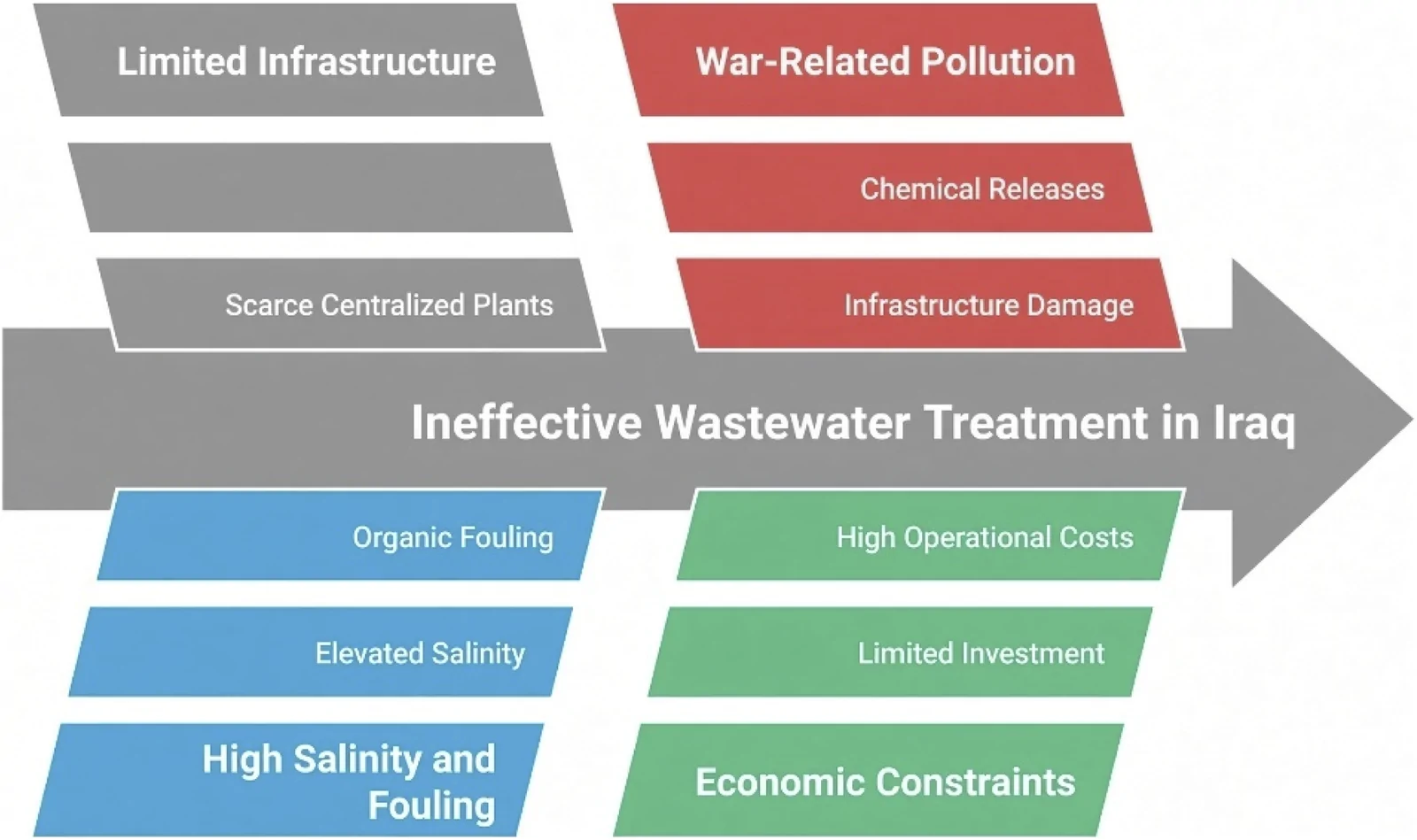
Overcoming wastewater treatment challenges in Iraq. Adapted from (ref. 32). Copyright © 2025, published by Polskie Towarzystwo Inzynierii Ekologicznej. Modification was made by the Gemini AI tool.

The main obstacles facing the application of adsorption technologies for treatment of contaminated water in Iraq are briefly described and illustrated in Fig. 8. High salinity conditions in Euphrates river water reduce adsorbent efficiency; hence, it is a major obstacle to achieving efficient heavy metal removal.^9,37,40^ Organic fouling by refinery and hospital effluents further complicates the process, since these sources of organic pollutants foul adsorbents, thus reducing their performance.^24,33,50^ Infrastructure has been damaged due to war-related pollution, especially in Mosul, which caused chemical releases and increased contamination challenges, with elevated lead (Pb) and cadmium (Cd) levels found in water sources.^27,39,43^ Infrastructure limitations rank first among other barriers because only about 30% of Iraq's population is connected to sanitary sewers; therefore, centralized treatment systems cannot be implemented effectively.^32,47^ Additionally, limited funds due to economic constraints hinder investing in large-scale treatment facilities, which may impede the smooth adoption of adsorption technology.^10,32,46^

### 7.2 Opportunities

**Fig. 9:**
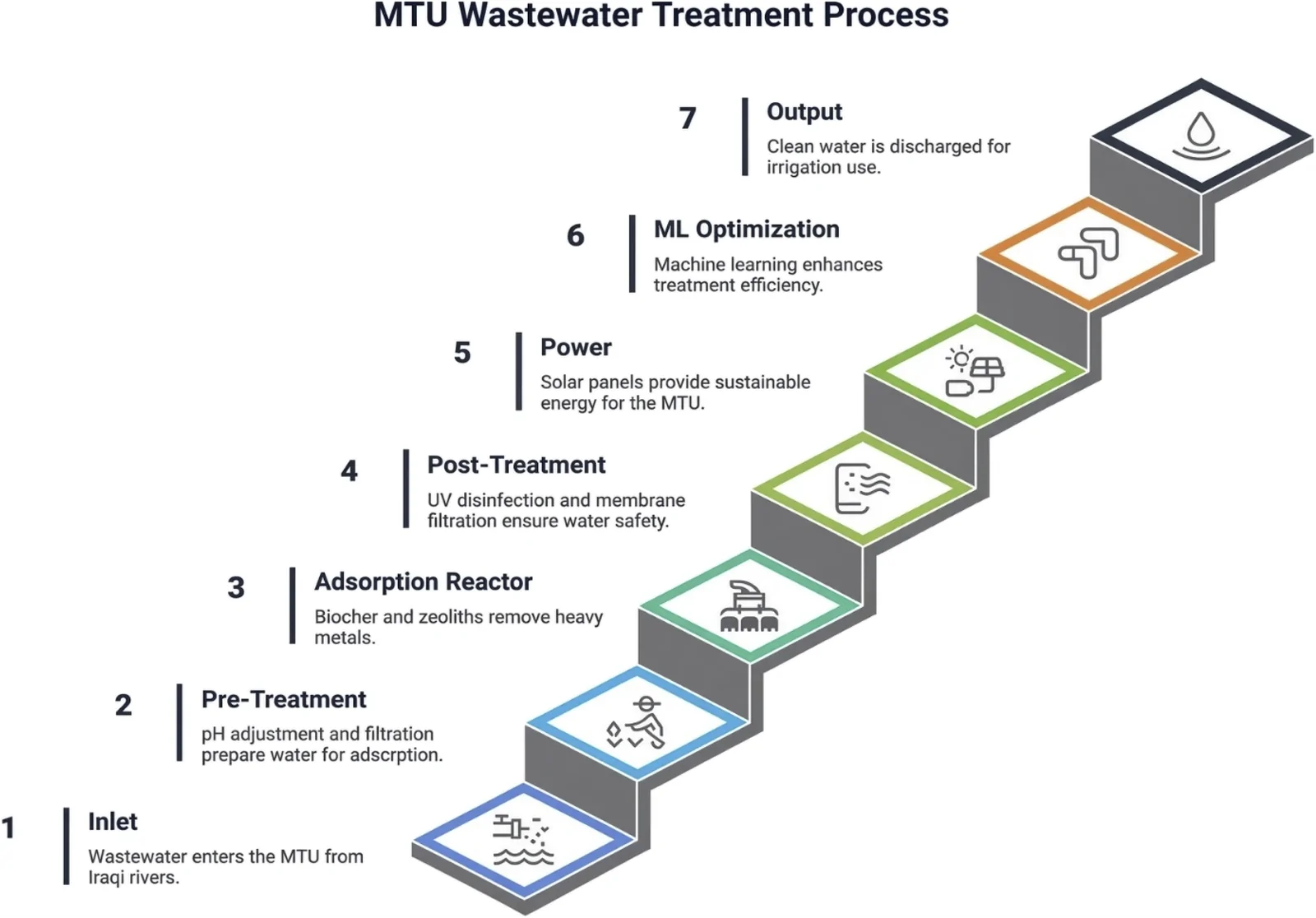
Schematic of Mobile Treatment Unit (MTU) for heavy metal removal in Iraq. Adapted from (ref. 48). Copyright © 2024, published by University of Baghdad. Modification was made by the Gemini AI tool.

The actual problems that Iraq faces regarding the application of adsorption technologies, and the great opportunities for innovative ways to tackle the mentioned limitations, are also discussed. Local adsorbents like rice husk biochar, date palm biochar, and Samarra zeolites will boost agricultural and mineral resources in Iraq since these resources significantly cut down the production cost of adsorbents.^5,17,34–36^ The heavy metal removal process adopts decentralization *via* Mobile Treatment Units in remote places with no proper infrastructure and in war-affected Mosul areas, using solar power and local adsorbents like rice husk biochar and Samarra zeolites. Fig. 9 shows the schematic design of an MTU, presenting its components and describing their function in Iraq's wastewater treatment.^15,48^ In addition, the system can be regenerated for up to five cycles of use; spent materials can be used as a soil amendment, thereby enhancing sustainability.^11,32,46^ Moreover, treated water can be recycled for irrigation, thus conserving water in places such as Al-Hizamat.^44,45^ Community empowerment training modeled after UNDP training will also build local expertise to strengthen capacity to manage and sustain such technologies.^47,49^

### 7.3 Implementation strategy

The successful implementation of advanced wastewater treatment strategies in Iraq requires a phased strategy that integrates technological demonstration, institutional collaboration, policy support, and data-driven process optimization.

• Pilot projects: testing MTUs in Baghdad, Basrah, and Mosul.^23,24,27,50^

• Partnerships: collaborating with USAID, UNDP, and SIGMA group for funding and expertise.^10^

• Policy support: advocating for subsidies and regulatory frameworks.^9,32^

• Monitoring: using ML for real-time optimization.^48,55^

## 8. Future directions and research gaps

Despite recent advancements, significant gaps continue to hinder the practical implementation of these technologies in Iraq, particularly the scarcity of pilot-scale studies, long-term performance and stability data, and computational approaches specifically tailored to the Iraqi environmental and operational conditions.^5,36,50,64^ Accordingly, the research agenda proposed in this study aims to address these identified gaps by promoting the development and application of locally available resources and technologies while aligning with the objectives of SDG 6, which focuses on ensuring the availability and sustainable management of water and sanitation for all.^91^

### 8.1 Pilot-scale validation

Pilot-scale validation is considered a critical milestone in the development of adsorption technologies for wastewater treatment applicable to varying Iraqi wastewater conditions. Such validation should initially be conducted in Baghdad using representative wastewater matrices.^15,23,48,50^ This approach is particularly important since most existing adsorption studies remain limited to laboratory-scale experiments and lack field-scale validation, thereby limiting their practical applicability and scalability.^9,44^ The proposed pilot-scale studies should test fixed-bed adsorption reactors packed with locally available rice husk biochar and zeolites using water from the Tigris river as a representative real-world matrix.^28,34,36^ Data on long-term performance, operational stability, regeneration potential, and treatment efficiency would help to bridge the gap between laboratory-scale research and practical field implementation. In the long run, such evidence may support the technical and economic assessment required to scale up these technologies and attract investment in sustainable wastewater treatment infrastructure in Iraq.^10,37^

### 8.2 Development of novel adsorbents

**Fig. 10:**
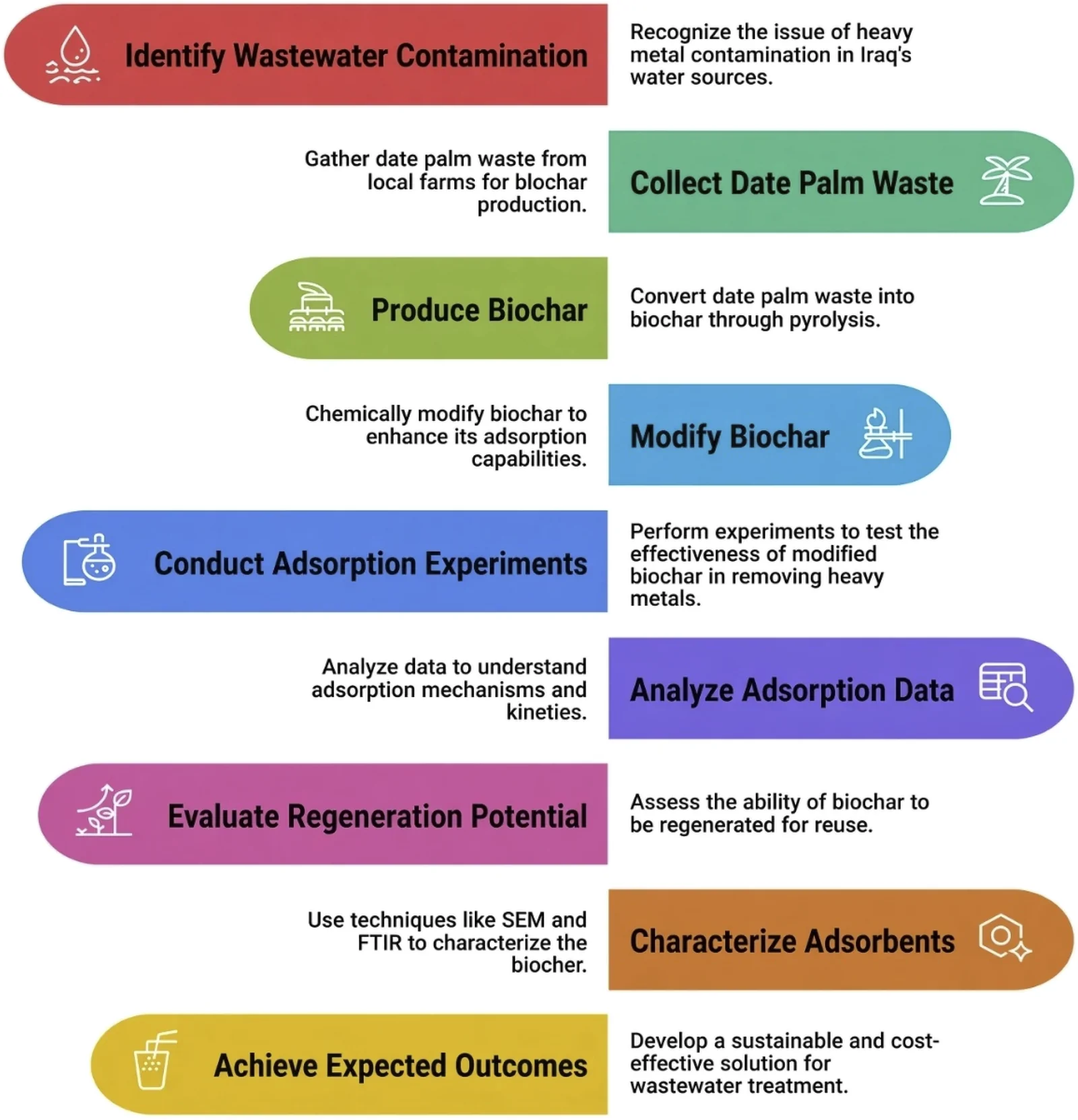
Development of novel adsorbents for wastewater treatment. Adapted from ref. 35. Copyright © 2025, published by Frontiers Media SA. Modifications were made using the Gemini AI tool.

One of the key elements of this thrust is to develop new adsorbents that modify local materials, such as date palm biochar, to achieve selectivity in a multi-metal system in highly saline wastewater.^35,40,51^ The basic rationale has been that new adsorbents are less efficient when exposed to complex matrices similar to those found in Iraqi wastewaters.^24,33^ This shall involve creating biochar with –COOH groups and testing its efficacy against Basrah refinery effluents. The issues shall be addressed through this performance testing.^45,46,57^ A product expected here would be low-cost, high-capacity adsorbents under very special Iraqi conditions.^5,11^Fig. 10 depicts the development route for these new adsorbents: functionalization and subsequent testing strategies to maximize their performance in such harsh wastewater conditions.^5,24,35,57^

### 8.3 Computational optimization

Computational optimization shall be the core of further development of adsorption technologies for wastewater treatment in Iraq, with plans to expand machine learning (ML) and molecular dynamics (MD) applications to predict long-term performance and optimize operational parameters.^55,56,59^ The rationale lies in the scarcity of computational studies specific to Iraq compared to the urgent need to improve process operations within the country.^10,68^ It thus proposes the development of Random Forest (RF) and Artificial Neural Network (ANN) models specific to high salinity conditions, together with MD simulations, for studying metal-adsorbent interactions at the molecular level.^15,57,58^ This approach can lower experimental costs while increasing efficiency, making it a scalable solution component for addressing Iraqi wastewater challenges.^9,49^

### 8.4 Computational models in multicomponent adsorption for real wastewater systems

Maximum adsorption capacities are the main criteria for ranking adsorbents in conventional adsorption studies. This parameter is useful for preliminary screening of materials but is not sufficient to reflect practical applicability because industrial wastewater treatment requires simultaneous optimization of adsorption efficiency, selectivity, regeneration ability, structural stability, operational cost, environmental sustainability, and technology readiness. Thus, the sole use of adsorption capacity frequently results in impractical material choices and poor transition from lab investigations to industrial implementation.^92–97^ Recent reviews also highlight that next-generation adsorption research needs to adopt integrated multi-criteria evaluation frameworks, instead of single-parameter comparisons, as exemplified in Tables 10 and 11. One of the key results of the proposed framework is the transformation of high-capacity adsorbents to deployable adsorbents. For example, graphene derivatives and Metal–Organic Frameworks (MOFs) normally have very high adsorption capacities. However, these materials have drawbacks for industrial use, such as high synthesis costs, difficulty in regeneration, poor hydrolytic stability, and limited scalability. In contrast, biomass-based adsorbents, natural zeolites, and hybrid biochar composites generally have lower maximum adsorption capacities but higher sustainability, economic feasibility, regeneration performance, and technology readiness, making them more attractive for large-scale environmental applications.^92,98–102^

**Table 10:** Qualitative multi-criteria evaluation of principal adsorbent categories for the elimination of heavy metals

Adsorbent family	Adsorption potential	Intrinsic selectivity	Regeneration and reuse	Structural/aqueous stability	Relative material and processing cost	Sustainability potential	Practical maturity	Reference
Rice husk biochar	Medium-high; highly dependent on pyrolysis and modification	Low-medium in pristine form; may improve after functionalization	Medium; regeneration performance varies with metal and modification	Medium-high, although ash release and long-term stability require assessment	Low	High when produced locally from agricultural residues	Medium-high	102–104
Date-palm-waste biochar	Medium-high; improved considerably by mineral or Fe modification	Low-medium in pristine form; medium-high after targeted modification	Medium; repeated-cycle evidence is less extensive than for activated carbon	Medium	Low in date-producing regions	High because it valorizes locally abundant waste	Medium	103 and 105
Natural zeolite	Medium; governed by mineralogy and cation–exchange capacity	Medium-high for many cationic metals, but lower for some oxyanions unless modified	Medium-high; desorption and ion-exchange regeneration are feasible but may not restore full capacity	High	Low	High where local deposits are available and minimal modification is required	High	106 and 107
Bentonite and related clay minerals	Medium; can increase substantially after chemical or composite modification	Low-medium in unmodified clay; modification may introduce target-specific sites	Medium	Medium-high; swelling and fine-particle separation may complicate operation	Low	High because clays are abundant and require relatively simple processing	Medium-high	108 and 109
Activated carbon	High because of its developed porosity and surface chemistry	Low-medium without targeted functionalization	Medium-high; thermal, chemical and electrochemical regeneration are possible, but may consume energy and reduce capacity over cycles	High	Medium-high	Low-medium; strongly dependent on precursor, activation route and regeneration	High	110–112
Graphene oxide and GO composites	High-very high in many laboratory systems	Medium; potentially high after functionalization	Low-medium; recovery, aggregation and structural changes may hinder repeated use	Medium; affected by pH, ionic strength, reduction and aggregation	High	Low-medium because synthesis, separation and possible nanomaterial release remain concerns	Low-medium	113 and 114
Metal–Organic frameworks (MOFs)	High-very high due to large accessible surface areas and tunable sites	High when pore chemistry is designed for the target ion	Medium; regeneration is feasible for selected MOFs, but repeated-cycle performance varies	Low-high depending on the framework; water stability cannot be assumed for the whole class	High	Low-medium because ligand synthesis, solvent use and scale-up remain important burdens	Low-Medium	115 and 116
Magnetic composites	High when magnetic recovery is combined with suitable active groups	Medium-high after functionalization	High in terms of separation convenience; chemical regeneration still depends on the binding mechanism	Medium-high, although oxidation, leaching and particle aggregation must be assessed	Medium-high	Medium; easier solid–liquid separation is advantageous, but nanoparticle production adds impacts	Medium	117 and 118

**Table 11:** Suggested framework for comprehensive decision-making in the selection of intelligent adsorbents

Decision criterion	Engineering objective	Primary evaluation parameter	Recommended computational support	Reference
Wastewater chemistry	Matrix characterization	pH, salinity, competing ions, natural organic matter (NOM)	Geochemical speciation modelling (PHREEQC) + Molecular Dynamics (MD)	119–121
Target metal	Adsorbent specificity	Pb, Cd, Cr, As, Ni, Cu; oxidation state; coordination chemistry	Density Functional Theory (DFT)	79 and 82
Adsorption mechanism	Mechanistic interpretation	Ion exchange, surface complexation, precipitation, electrostatic attraction	DFT + explicit-solvent MD (or AIMD)	114 and 120
Material selection	Sustainable adsorbent design	Capacity, selectivity, regeneration, stability, cost	Machine-learning (ML)-assisted screening	61 and 122
Scale-up	Industrial implementation	Technology readiness level (TRL), operating cost, environmental impact	AI-assisted optimization + techno-economic analysis (TEA) + life-cycle assessment (LCA)	123 and 124

Based on the comparative analysis presented in this review, adsorbent selection should follow a sequential decision-making scheme rather than rely solely on direct comparisons of adsorption capacities. The first step is to characterize the wastewater composition (salinity, pH, competing ions, NOM concentration, and dominant heavy-metal species). Subsequently, the adsorption mechanisms should be identified to determine the dominant mechanisms for pollutant removal, such as ion exchange, surface complexation, electrostatic attraction, precipitation, or redox reactions. Finally, computational screening by Density Functional Theory (DFT), explicit-solvent Molecular Dynamics (MD), and Machine Learning (ML) should be combined before pilot-scale validation. This type of workflow reduces the disparity between laboratory findings and engineering application, aligning well with the contemporary movement towards digitized wastewater management.

### 8.5 Policy and infrastructure integration

Beyond the efficiency of adsorbents, the implementation of adsorption technologies in Iraq is influenced by several practical challenges, including economic conditions, infrastructural limitations, and institutional support. In previously published studies, the need for effective regulatory policies and implementation strategies was emphasized to promote the adoption of adsorption-based water treatment technologies.^27,32^ Nevertheless, large-scale adoption is still limited by budget constraints and lack of wastewater treatment facilities, particularly in areas of long-term conflict and under-investment.^28,43^ With advances in technology, financial support programs and community-level awareness campaigns may help promote local participation and increase technical capacity for wider application.^47^ Tackling these challenges would not only advance SDG 6 (Clean Water and Sanitation) and promote more sustainable wastewater management in Iraq, but also improve the long-term profitability of adsorption technology.^46,91^

## 9. Case studies and practical applications

This section focuses on the use of adsorption techniques in Iraq and presents case studies from Baghdad, Basra, and Mosul. It details the application of these approaches, the results obtained, the challenges faced, and the lessons learned.

### 9.1 Case study: Tigris river (Baghdad)

The Tigris river in Baghdad is heavily polluted by hospital effluents and urban discharges, with reported Pb concentrations of 20–100 µg L^−1^ and Cd concentrations of 5–30 µg L^−1^; both exceed WHO standards for safe drinking water, which is of considerable concern.^5,23,26,125^ Most notably, this has been attributed to Al-Kindi Hospital contributing a large load of Pb, thereby aggravating the already existing problem of contamination.^47,126^ Recent attempts, including a 2023 pilot study, have set up a fixed-bed reactor using rice husk biochar to treat about 500 L per day of Tigris water at pH 5 and a temperature of about 25 °C, with a flow rate of approximately 10 mL min^−1^, yielding an estimated removal efficiency for Pb of about 92% within two hours.^10,11,34,50,127,128^ It produced effective results, achieving high removal rates for Cd as well. This will reduce operational costs by 30%, illustrating the cost-effectiveness of wastewater treatment when implemented, particularly in urban settings.^55,56,129^ Results of the pilot seem encouraging; three cycles of biochar remain efficient before fouling occurs, with about 15% reduced performance, still yielding water that meets WHO lead standards (<10 µg L^−1^), which can then be reused for purposes such as irrigation.^7,50,130,131^ The issues subsequently transition to organic fouling caused by hospital effluents, necessitating pre-treatment through coagulation–flocculation to ensure the continued effectiveness of the adsorbent.^5,132^ There has been an expansion of infrastructure to accommodate larger volumes, thereby requiring facility enhancement.^32,52^ Key insights include the importance of pretreatment and machine learning optimization for effective urban wastewater management, as well as community training to sustain operations within local capacity.^47,133^

### 9.2 Case study: Euphrates river (Basrah)

The Euphrates river in Basrah receives a large amount of oily waste from nearby refineries. The salt content is high (1000–3000 mg L^−1^), and there's an elevated level of lead (Pb) as well (between 50–150 µg L^−1^). Al-Dora refinery also releases a certain amount of Cadmium (Cd) and Mercury (Hg).^24,30,31,37,40,133^ In the pilot study carried out in 2024 to address this issue, a mobile treatment unit based on date palm biochar was used to treat 1000 L per day of river water at pH 5.5 and 30 °C, achieving about 88% removal of Pb and 82% removal of Cd.^15,17,35,48,134,135^ Molecular dynamics simulations guided the functionalization of biochar and increased the efficiency of binding capacity for Pb by about twenty percent, hence showing the potential for high-level computational tools during optimization studies for adsorbent performance.^56,57,136^ The pilot study results were encouraging since the MTU-treated water achieved a Pb level below 15 µg L^−1^, which would otherwise make it suitable for reuse in agriculture.^46,137^ Regeneration of the adsorbent extended biochar's lifetime to four cycles, thereby improving sustainability.^11,138^ Challenges encountered included a 10% reduction in efficiency after three cycles due to high salinity, thus requiring a desalting pre-treatment step to maintain efficiency.^40,139^ This will elevate the operational costs of the facility due to its reliance on solar energy; consequently, this may pose a constraint when attempting to scale up.^48,140^ Lessons learned herein demonstrate that functionalized biochar and hybrid systems operate most effectively under saline conditions. Consequently, subsidies can be considered as viable strategies to offset the operational costs of an MTU when these systems are implemented more broadly in challenging environments such as Basrah.^32,141^

### 9.3 Case study: Mosul (war-affected area)

Groundwater and Tigris river contamination with chemicals is very high in Mosul, an area affected by war. Every crisis increases the levels of metals such as Cr by 50–200 µg L^−1^ and Pb by 50–150 µg L^−1^ because post-conflict debris exacerbates it.^27,39,42,43,142^ MTU was piloted with Samarra zeolites for a proposed 2024 trial operation to treat groundwater at pH 4.5, achieving 90% Cr removal and 85% Pb removal at a rate of 800 L per day.^5,28,36,48,143,144^ In full-potential manifestation toward achieving real-time decentralized treatment systems in difficult environments, as demonstrated by the MTU ML model flow optimization for lowering energy consumption rates, this optimization reduced actual energy consumption by up to one-fourth.^55,56^ The trial was successful, as treated water complied with WHO standards for chromium (<50 µg L^−1^), supporting the community's water supply.^9,145^ Notably, zeolites were regenerated for three cycles, adding some more sustainability to the process.^36,146^ The drawbacks noted included reduced efficiency due to debris and organic pollutants that would require filtration pre-treatment.^50,147^ A lack of local technical expertise poses a significant challenge, particularly for equipment operation and maintenance, necessitating capacity-building and technical training programs.^47,148^ Lessons learned are that MTUs work well in decentralized remediation in war-affected areas like Mosul but require robust training programs to ensure operational sustainability and long-term success.^32,149,150^

## 10. Emerging trends and breakthroughs in adsorption technology

**Fig. 11:**
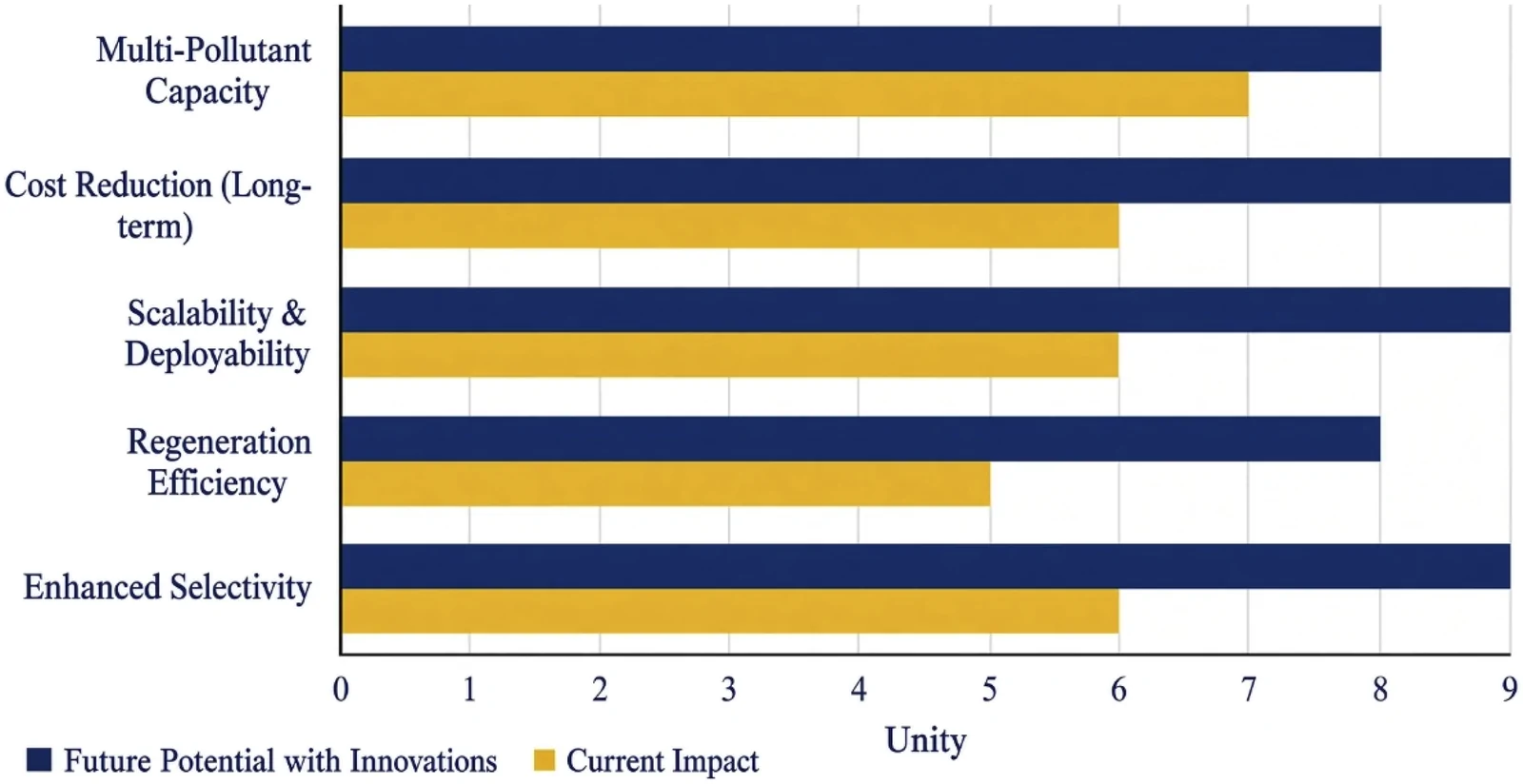
Projected impact of future innovations on the performance of adsorption technologies for heavy metal elimination in Iraq. Adapted from (ref. 57). Copyright © 2024, published by Elsevier. Modification was made by the Gemini AI tool.

The field of adsorption, therefore, can be considered fast-evolving, with high hopes of leveraging recent innovations to improve water treatment in Iraq.^1^ Ongoing advancements include the development of smart materials, which can evolve into adaptive variants.^15^ This involves integrating sensors with adsorbents to enable water-quality monitoring and automated regeneration, which would improve efficiency and sustainability. Improving adsorption capacity, establishing breakthrough time, and designing highly specific adsorbents for the major pollutants found in Iraq (such as heavy metals mainly found in industrial effluents) could advance the field. Molecular modeling and the incorporation of artificial intelligence could make this possible.^18,55,57^ A membrane-adsorption hybrid system is an emerging protocol that combines adsorption and membranes to produce high-quality water and improve operational efficiency, especially in industrial effluent treatment in Iraq.^9^ These innovations are likely to significantly enhance adsorption-based technologies for heavy metal remediation. Incorporating MD and AI may provide exceptional accuracy in sorbent design, while smart materials can support real-time process control.^12,56^ The bar chart in Fig. 11 illustrates the anticipated impact of smart and adaptive materials, molecular modeling and AI, and membrane-adsorption hybrid systems on heavy metal removal in Iraq, indicating a substantial enhancement of water treatment capabilities.^55,57^ It can therefore be concluded that these novel technologies have considerable potential to improve water quality in Iraq by reducing heavy metal contamination while aligning with the sustainable development goals.^91^

### 10.1 Adsorbent selection decision matrix

The conceptual frameworks presented in this review, compared to conventional review articles that summarize the reported adsorption performances, were developed by incorporating engineering feasibility, sustainability, regeneration potential, and economics into an integrated decision support strategy. These frameworks are developed to help researchers to choose suitable adsorbents by taking into account the requirements of practical applications rather than just the adsorption capacity. Table 12 is a summary of an application-oriented decision matrix between treatment scenarios and appropriate adsorbent families that can be used to operationalize the proposed conceptual framework into practical material selection guidelines. This framework integrates adsorption performance with cost, regeneration potential, structural stability, selectivity, and operational feasibility, which is different from descriptive summary tables.

**Table 12:** Application-oriented adsorbent selection framework linking wastewater-treatment scenarios with suitable adsorbent families and dominant engineering selection criteria

Application scenario	Recommended adsorbent family	Primary selection criterion	Scientific justification	Reference
Low-cost rural or decentralized water treatment	Biochar/biomass-derived adsorbents	Low cost, local availability, sustainability	Suitable when cost and simple preparation are more important than maximum capacity	151 and 152
Industrial wastewater treatment	Activated carbon/zeolite	Stability, regeneration, operational reliability	These materials are structurally stable and more compatible with large-scale treatment systems	153 and 154
Selective recovery of valuable or specific metals	Functionalized MOFs	High selectivity and tunable binding sites	MOFs offer tunable porosity, functional groups, and high affinity toward targeted metal ions	155 and 156
Multi-component wastewater containing competing ions	Hybrid/composite adsorbents	Multiple adsorption mechanisms and improved selectivity	Hybrid systems can combine ion exchange, complexation, electrostatic attraction, and surface precipitation	157 and 158
Continuous fixed-bed or column operation	Zeolite/activated carbon/mechanically stable composites	Mechanical strength, durability, and regeneration	Continuous systems require adsorbents with stable particle structure, low attrition, and repeated usability	159 and 160
Rapid separation after treatment	Magnetic composites	Easy recovery and reduced filtration demand	Magnetic adsorbents simplify solid–liquid separation and can improve reusability	161
Industrial wastewater containing recalcitrant pollutants and mixed heavy metals	Functionalized biochar–metal oxide nanocomposites	High adsorption efficiency, multifunctionality, and reusability	Hybrid biochar–metal oxide nanocomposites combine adsorption with catalytic activity and enhanced regeneration, making them promising candidates for treating complex industrial wastewater	162
High-strength industrial wastewater requiring advanced treatment	Functionalized biochar composites	High stability and broad-spectrum pollutant removal	Recent developments demonstrate that functionalized biochar composites provide improved structural stability, abundant active sites, and enhanced removal of diverse contaminants compared with conventional adsorbents	162

## 11. Conclusion

Severe heavy-metal pollution in Iraq's two major waterways, the Tigris and Euphrates rivers, demands an effective remediation strategy. This study critically discusses recent advances in adsorption technologies for heavy-metal removal, addressing practical issues such as saline water, coexisting organic pollutants, and the use of locally accessible, sustainable adsorbents. The focus extends beyond adsorption capacity to include regeneration, stability, scalability, and real-world application. The study also stresses the increasing importance of computational approaches in providing mechanistic insights and supporting reasonable adsorbent material selections for future water-treatment applications. The key results of this assessment are:

• Rice husk biochar and zeolites from Samarra are among the regional adsorbents that can achieve excellent removal efficiencies (>90%) for lead, cadmium, and chromium under optimized conditions.

• Machine learning algorithms are useful tools to improve the selection of the process and material because they can predict the efficiencies of adsorption with an accuracy of more than 95%.

• Molecular dynamics (MD) simulations give key insights for the rational design of advanced adsorbents through unveiling the atomic-scale adsorption mechanisms.

• The substantial challenges, accompanied by specified remedial measures, include the high salinity levels of the Euphrates river (ranging from 1000 to 3000 mg L^−1^) and the deficiency of adequate sanitation infrastructures, with merely 30% of Iraq's population having access to sewage networks.

• Adsorption technology is cost-effective and can be easily scaled up. It is a more durable solution than the previous methods. It is consistent with the principles of the circular economy because it uses by-products from agriculture and manufacturing.

Additionally, this review described an integrated approach to enhance heavy-metal contaminant removal from water in Iraq by combining adsorption technologies with computational modelling. Instead of viewing experimental optimization and molecular simulations as distinct methods, the framework demonstrates how they can complement each other and serve as a foundation for choosing appropriate adsorbents based on local environmental conditions. The methodology developed can reduce experimental effort, improve process design, and facilitate the development of more reliable treatment systems. Notably, this work also contributes to the objectives of Sustainable Development Goal 6 by promoting research to increase access to safe and sustainable water treatment technologies.

## Conflicts of interest

The authors declare no conflicts of interest.

## Data Availability

The authors declare that no primary research results, software, or code have been included, and no new data were generated or analyzed as part of this review.
